# An Interactive Process for Delivering Pharmacologic Interventions for Migraine Headache to First-Year Medical Students

**DOI:** 10.15766/mep_2374-8265.10877

**Published:** 2020-02-07

**Authors:** Jennifer Cleveland, Renée J. LeClair

**Affiliations:** 1 Assistant Professor, Department of Basic Science Education, Virginia Tech Carilion School of Medicine; 2 Associate Professor, Department of Basic Science Education, Virginia Tech Carilion School of Medicine; 3 Chair, Department of Basic Science Education, Virginia Tech Carilion School of Medicine

**Keywords:** Migraine, Migraine Disorders, Headache, Active Learning, Case-Based Learning, Flipped Classroom

## Abstract

**Introduction:**

This interactive didactic session is designed for first-year medical students to explore the common clinical symptom of headache and its various management strategies. The session provides an opportunity to cover a variety of drugs, mechanisms of action, drug-drug interactions, and routes of administration in a single 50-minute time frame.

**Methods:**

Using a modified case-based approach, we designed an interactive session for 41 first-year medical students. Students prepared for the session using basic learning objectives and a table of drugs that treat headache pain. In class, we distributed a patient scenario and a series of discussion questions to explore headache management. We assessed student performance using questions purchased from the National Board of Medical Examiners and student perceptions using both qualitative and quantitative data collected from faculty and end-of-block evaluations.

**Results:**

Student performance on purchased questions related to content was significantly increased when compared to the national average (*n* = 5; 90.6% ± 6.0% vs. 82.6% ± 8.5%; *p* = .0052). Student perceptions of the overall quality of the faculty, content presentation, and material were positive (4.4 out of 5.0). Two themes emerged in the end-of-block evaluations: Students commented positively on the prereading materials, and students commented on the need to address underlying physiology associated with the discussed pharmacology.

**Discussion:**

This flexible activity can be delivered in a short time (50 minutes) by a single faculty member in a variety of curricular structures. Our data demonstrate strong student performance and suggest that incorporating additional content would enhance delivery.

## Educational Objectives

By the end of this activity, learners will be able to:

1. Describe the therapeutic uses, adverse effects, toxicities, and contraindications of agents used for acute and prophylactic management of migraine.
2. Apply pharmacokinetic parameters and formulation to select migraine treatments.
3. Interpret information from clinical scenarios to identify agents used for nausea and vomiting associated with migraine.

## Introduction

In the US, severe headaches and migraine affect approximately 10% of males and 20% of females over a 3-month period.^1^ This common presentation represents a significant financial burden to both the individual and the health care system through both direct and indirect costs.^2,3^ Although nearly all individuals will experience this presentation over their lifetime, delivery of content related to mechanisms of migraine and its management remains limited in undergraduate medical education.^1,4^

The American Headache Society has been a strong advocate for introducing headache education in medical schools; however, current surveys of undergraduate medical education programs still report 20% of programs with no formal coverage of the content topic.^5^ There is some evidence to suggest that headache is covered more generally as a subtopic of pain or acute pain, but there are few data describing the amount of time or emphasis this specific content is given among other pain presentations.^6^ The general consensus across many organizations^4–7^ is that regardless of whether headache is delivered independently or in conjunction with larger curricular elements, the emphasis on this common presentation is lacking despite the overarching clinical implications of migraine pain.

In our current program, headache pain is briefly addressed as one of the many acute pain presentations. To enhance this coverage, we implemented the session described here to address management of headache and migraine pain. During this time frame, the pharmacology content at Virginia Tech Carilion School of Medicine (VTCSOM) was also undergoing a revision to optimize curricular time and address student performance. Given the need for migraine education and our curricular renovation of pharmacology, we took this opportunity as a starting point to illustrate various treatment options in a modified case-based setting.

The activity presented here is targeted to first-year students and designed to be an interactive lecture with concepts placed in the context of a clinical case. We have designed the class session using a modified version of the five S's^8^ (significant, same, simultaneous, specific, and summarize). This is a modified version of the process well documented for team-based learning,^9^ with *summarize* added as the fifth S. Briefly, we use a single case scenario representing a *significant* clinical problem to introduce the session. Following the case, we present a series of 12 questions, and we ask students to work on one question at a time to focus everyone on the *same* task. These questions generally address several of the subcompetencies of the prescribing process,^7^ including choosing the drug, dosing and frequency, duration of therapy, and reviewing after additional information has been provided. The activity focuses less on making a diagnosis, prescription writing, and informing the patient, as these subcompetencies are addressed in the Clinical Skills portion of our curriculum. Following each question, students discuss their answers in the large group, allowing answers to be reported *simultaneously* and *summarized* before moving forward. A few questions require *specific* choices; use of this format was a concerted decision. To allow for flexibility of topical discussion, multiple-choice questions have not been implemented extensively. Using this generalized questioning approach allows us to rapidly update the activity as new therapeutic guidelines, recommendations, or contraindications arise. Similar case-based strategies have been well documented and were an influencing factor in the development of this activity and new curricular pharmacology structure.^10,11^

Finally, curricular struggles with pharmacology content and student performance are common to many programs.^12,13^ Despite this challenge, there are few published activities to assist with effective delivery. The few *MedEdPORTAL* publications on headache and migraine either (1) are not clinically current,^14,15^ reflecting the ever-changing nature of pharmacologic disease management, or (2) focus on M3-M4 learners in a clinical OSCE setting.^16^ Thus, this resource fills a gap for both first-year pharmacology content and connections to contemporary migraine headache management current with practice guidelines. It also allows for a flexible format that can readily be updated from year to year with minimal modifications and incorporated as an activity into a variety of curricular formats.

## Methods

At the time of the activity, VTCSOM had a class size of 41 students per year, and the curriculum was divided into four main disciplinary domains: Basic Science, Clinical Skills, Research, and Interprofessionalism. The content in each of these domains was delivered across four 10-week blocks in the first year in an organ systems–based approach. Pharmacology content was delivered within the Basic Science domain, which included large-group (total of 9 hours per week for all basic sciences) and problem-based learning sessions. We delivered this learning activity to first-year learners in the seventh week of an 8-week block covering the biology of the nervous system.

The case-based session was delivered in an active lecture format by a single faculty member (a pharmacist) in a traditional lecture-style classroom to approximately 20 first-year medical students over a 50-minute session. (Ideally, space better suited for group work would have been utilized.) The Migraine Facilitator Guide (Appendix A) summarized the specifics of the activity. Briefly, prior to the session, we asked students to review a chart containing drugs for migraine treatment^17^ along with associated session learning objectives (Appendix B: Advance Preparation Materials). These resources were posted on Blackboard at the start of the block and were available to the students at all times. Preparation materials were distributed to students at least 1 week in advance of the session. During the session, we asked students to work through the case in groups of two to three (Appendix C: Student Migraine Presentation). This resource was released on Blackboard immediately before the class session; students did not have access to the resource prior to class. Faculty used Appendix D to lead the classroom session. The session started by reminding students of the basic objectives (covered by the preclass preparation), followed by the applied learning outcomes for the session. After this introduction, we presented a case scenario followed by a series of 12 questions that allowed students to apply their basic understanding of drug classes, mechanism of action, drug-drug interactions, and routes of administration (Appendix D). Students were asked to answer questions sequentially as they unrolled in the case presentation. We allowed approximately 2 minutes per question for student groups to research the topic and determine answers. Following the research time, we randomly chose student groups to share their answer with the whole class. We followed up by summarizing the best approach to address the question before moving on to the next question (or set of questions). Question summaries were limited to approximately 2 minutes per question to stay on track and complete the exercise in the 50 minutes allocated. Appendix D was posted at the end of the activity for students to use as a resource.

We assessed the activity using NBME questions purchased for the end-of-block summative examinations; content was assessed at the end of the block and again at the end of the students' second year. Performance on five NBME questions that related to the content covered in the activity was compared to the mean national performance. Qualitative and quantitative feedback from open commentary on both faculty evaluations and end-of-block evaluations was used as a measure of student satisfaction and overall effectiveness of the activity.

## Results

The activity was delivered one time during Block IV of the first year, and students were assessed on the same content at two intervals. Approximately 50% of students in the first-year class at VTCSOM participated. This attendance was typical for our program; however, all students had access to the session materials and class recordings. The Block IV examination consisted of 152 questions spanning the physiology and anatomy of the central and peripheral nervous system, basic neuroscience, and neuropharmacology; all 41 students completed the assessment. The second assessment of this material occurred in Block VIII (final course) of the students' second year. Student performance on the five questions related to content delivered in the migraine case-based activity was significantly different from the national average (*n* = 5; 90.6% ± 6.0% vs. 82.6% ± 8.5%; *p* = .0052, one-tailed *t* test).

Qualitative and quantitative metrics from the faculty evaluations are summarized here. Faculty and end-of-block evaluations were completed by all 41 students. The faculty member was evaluated by all students using five Likert-style questions (0 = *strongly disagree*, 5 = *strongly agree*) and four open-ended questions. Students responded positively in all aspects evaluated, scoring 4.4 or above out of 5.0. In response to “This faculty member appropriately organized the content, flow and pace of the presentation” and “I found the materials provided to be effective learning tools,” students responded favorably (4.4 and 4.5 out of 5.0, respectively). In response to the open-ended question “What did you find effective about the materials?”, a positive student perception of the posted prereading materials emerged as a theme, suggesting the concise table was a realistic amount of preparation for a 50-minute session (four out of eight comments). An example comment was “The drug charts were so helpful!” In response to the open-ended question “What could be done to improve the lectures?”, students commented on a deficit in underlying neurophysiologic concepts needed to fully grasp the mechanism of actions of the drugs discussed (four out of seven comments). An example student comment was “Spent little time talking about the underlying physiology and I think that made it harder to see how the drugs are working.” No negative commentary on the session itself was reported.

## Discussion

Despite a limited assessment tool (five NBME questions), we were able to illustrate enhanced student performance on the content delivered exclusively in this 50-minute session at two different time intervals. These scores were significantly different from national averages, representing an overall increase in performance on pharmacology-based content for our students. Students had a positive perception of the overall flow and organization of the content and found the materials to be effective learning tools. Based on these results, we will likely use a similar classroom model to develop sessions on other pharmacology topics. These active sessions can easily be generated using tables from existing textbook resources as preparation material and short case scenarios followed by a series of questions similar to those presented here based on the pharmacology subcompetencies.^7^

Quantitative and qualitative student comments highlighted some key aspects of active classroom sessions and the integrated curriculum. First, oftentimes, faculty perceive the need to reinvent the wheel when it comes to development of prereading or in-class materials. In reality, a wealth of resources is available through textbook publishers, journals, and even YouTube that can be adapted to suit individualized needs and save time. In this case, a single faculty member was able to adapt available resources (e.g., tables from *Lange Smart Charts: Pharmacology*^17^) to generate focused preparation materials that supported the learning activity. Students commented on this resource as a positive aspect of the session and their ability to prepare for the class. Second, in an integrated curriculum such as ours, it is important to know what has been presented previously to build on student knowledge. The activity presented here was clinically based and effective at engaging the students; however, students suggested that they did not have a sufficient understanding of the physiologic mechanisms targeted by the drugs discussed. As noted previously, headache was addressed in conjunction with other acute pain presentations; however, students did not feel that this prepared them well for a session focusing on headache management, which may highlight an additional need in the curriculum. As a result, we likely will revisit how headache pain is delivered in future iterations. On a positive note, this student feedback highlights the potential for in-class integration during the session, and future adaptations of the session could incorporate the mechanisms of headache pain. It is possible that the incorporation of a physiologist during this 50-minute session may be sufficient to address any gaps in underlying basic neuroscience mechanisms needed to determine the appropriate mechanism of action. Regardless, it is important for individuals implementing this activity to be sure to consider the background knowledge of their learners.

As the assessment questions and individualized student performance are not readily available from the NBME, these data should be interpreted with caution. There is a limited assessment set from which to draw on the efficacy of the activity, and adding a more comprehensive set of knowledge questions will gather more evidence about the impact of this work. In the future, we may consider adding a pre- and postassessment of the students in the classroom to obtain additional data about the changes in student knowledge. The students' overwhelmingly positive perceptions of the activity and ease of development are still valuable aspects of the activity that should not be overlooked. In summary, this activity represents a flexible method for pharmacology delivery that can be adapted to many different topics and curricular styles with positive student perception.

## Appendices

A. Migraine Facilitator Guide.docx
B. Advance Preparation Materials.docx
C. Student Migraine Presentation.pptx
D. Facilitator Migraine Presentation.pptx All appendices are peer reviewed as integral parts of the Original Publication.
