# Supplementary material for: An Interactive Process for Delivering Pharmacologic Interventions for Migraine Headache to First-Year Medical Students
Source: MedEdPORTAL. 2020 Feb 7;16:10877. doi: 10.15766/mep_2374-8265.10877 (PMC7012313; doi:10.15766/mep_2374-8265.10877)
Supplement: Supplementary file 1 — A. Migraine Facilitator Guide.docx B. Advance Preparation Materials.docx C. Student Migraine Presentation.pptx D. Facilitator Migraine Presentation.pptx [file mep-16-10877-s001.zip › A. Migraine Facilitator Guide.docx]

**Facilitator Migraine Treatment Guide**

**Description of the resources needed for this activity**

1. Facilitator Migraine Treatment Guide.doc – This document contains all the materials needed for an individual faculty member to conduct this activity.
2. Advance preparation materials for students.doc – This document references a pre-reading chart for students to become familiar with drug names, drug class, and brief description of mechanism of action prior to class. This is posted should be distributed to students in advance of the session (at least 1 week prior).
3. Student Migraine Treatment PowerPoint.ppt – This student slide deck is released to the students immediately before class and contains only the cases and associated questions.
4. Facilitator Migraine Treatment PowerPoint.ppt – This slide deck contains all content questions, answers and figures to facilitate the large group discussion. This is released to the students after the conclusion of the session.

**Outline of the in-class activity:**

Prior to arriving:

- Students should review objectives listed below and the table referenced in Appendix B^1^: Advance preparation materials for students.doc, to acquaint themselves with drug names, drug class, and brief description of the mechanism of action. This preparation should take the students less than 15-minutes.
  - Learning objectives:
    - Identify classes of medications utilized for migraines.
    - List agents within each class and recognized drug name endings associated with these agents.
- For general information for of headache management the facilitator can refer to ^2,3,4,5^ . Additional context specific references are added throughout this document and can be referred to for discussion.

During the 50-minute activity:

- At the time of class, Appendix C: Student Migraine Treatment PowerPoint.ppt is released for the students to follow along with the activity.
- Faculty lead the discussion using Appendix D: Facilitator Migraine Treatment PowerPoint.ppt.
- The slides consisted of a single case presentation followed by a series of questions (1-12) and the students were asked to address these questions in small groups.
- The questions unroll in the student PowerPoint one or two at a time.
- To keep the activity moving rapidly, the facilitator summarized key points of questions on each slide before moving forward and asking the students to address subsequent questions.

The breakdown of the questions and discussion points on the slides is outlined below. While the students research their answer, the facilitator should circulate amongst the groups, listening and asking questions to help guide students toward important points. The facilitator can then call on a group to share and explain their answer. Following this process, faculty would choose groups of students to explain their answers. When students present the answer, it is advisable to elicit their reasoning, providing confirmation or correction as appropriate.

**Appendix D: Facilitator Migraine Treatment PowerPoint.ppt**

Description of narrative for each of the slides in Appendix D.

**Slides 1 – 2:**

- Faculty facilitator introduces the activity and the in-class learning objectives.
- Students are asked to work in groups of 2 to 3 for the next part of class. Using Appendix C, students will work through case scenario and questions. Students can use any resources they see as necessary. It is important to note, that students should work on a single question at a time, or a group of questions presented together, in the order in which they appear in the PowerPoint.
- Questions are presented either individually or several on a single slide and students are allotted ~2 minutes to arrive at an answer in their group.
  - The questions vary in difficulty and discussion time can vary accordingly.
  - Questions are summarized by the faculty before students can move on to the subsequent questions. Summary should not take longer than 2 minutes a question

**Slide 3:**

- Use this slide to introduce the case.
- Ask the student groups to answer the first 2 questions.
- After ~4 minutes choose a group to answer question 1.
- Engage the class in discussion before moving on to Slide 4.

**Case:**

Eddie is a 40-year-old female who comes into your clinic after having to take another day off from work. She is a lawyer and typically works more than 50 hours a week in addition to her responsibilities at home. Eddie’s headaches have been severe with increasing frequency over the past few months and are preceded by a feeling of lightheadedness and visual disturbances (disturbed vision, flashing light). The pain she describes is unilateral and is accompanied by severe nausea. Her self-treatment with naproxen 220 mg has not provided much relief.

*Questions 1 and 2:*

1. What is the mechanism of action of Nonsteroidal Anti-Inflammatory Drugs (NSAIDs)?
2. What are the adverse effects of NSAIDs which necessitate patient counseling?

**Slide 4:**

Use this slide to summarize the answer to question 1.

*Summary of question 1:*

1. What is the mechanism of action of NSAIDs?

- Points of discussion:
- NSAIDS reversibly inhibit the enzyme cyclooxygenase
- Inhibition is at both COX-1 & COX-2
- This inhibition leads to a reduction in prostaglandin synthesis
- The physiological impact is a generalized anti-inflammatory and analgesic action
- For more information see^6^.

**Slide 5:**

- Choose a student group to answer question 2.
- Engage the class in discussion. The slide is animated so the facilitator can engage the students in discussion and summarize the answer to question 2 before moving on to slide 6

*Summary of question 2:*

1. What are the adverse effects of NSAIDs which necessitate patient counseling?

- Adverse Effects:
- Gastrointestinal (GI) effects include: dyspepsia, nausea, abdominal pain, diarrhea, ulcer
- COX- 1 inhibition suppresses mucosal cytoprotective prostaglandins, local administration leads to GI irritation
- Cardiovascular complications arise from selective suppression of COX-2, leaving COX-1 functioning
- Decreased prostaglandin synthesis results in decreased renal blood flow and potentially acute kidney injury.
- Use caution in patients with the following comorbidities:
  - Impaired renal function,
  - Heart failure
  - Patients taking diuretics
  - ACE inhibitors
  - Elderly

**Slide 6:**

- Ask the student groups to answer questions 3 -5.
- After ~6 minutes choose a group to answer question 3.
- Engage the class in discussion before moving on to Slide 7.

She (Eddie) has also tried taking 1 tablet of Acetaminophen 250 mg/aspirin 250 mg/caffeine 65 mg (Excedrin Migraine) which is only a little more effective.

*Questions 3, 4 and 5:*

3. Is there benefit of this over-the-counter (OTC) treatment?

4. How does aspirin differ from other NSAIDs?

5. How could the medications be administered differently to increase their effectiveness?

**Slide 7:**

- Use this slide to summarize the answers to questions 3 - 5.
- The slide is animated so the facilitator can reveal a question, engage the students in discussion and then summarize the answer for questions 3-5.

*Summary of questions 3 and 5 (text is animated so each question can be discussed separately):*

1. Is there benefit of this over-the-counter (OTC) treatment?
   - It is important to discuss that ~71% of patient’s self-treat with OTC products before seeking additional help.
   - The combination of drugs is proposed to work in the following manner:
     - 1) Acetaminophen is a mild COX inhibitor
     - 2) While aspirin is a more effective COX inhibitor (suicide inhibitor)
     - 3) Caffeine is believed to constrict cerebral blood vessels and may increase absorption of other agents ^7^.
   - This combination of drugs has been shown to be helpful for pain and inflammation.
   - Despite its generalized use, there is a greater potential for over use and rebound headache when using OTC treatments alone in excess of 10 days ^8^.
     - Students may explore this topic and the facilitator could expand the discussion of MOH.
       1. Medication over use headaches (MOH) is a headache occurring on 15 or more days per month, developing as a consequence of regular overuse of acute or symptomatic headache medication for over 3 months. (Simple analgesics and NSAIDS – 15 days/month) (triptans, opioids, combination analgesics 10 days/month) ^9^.
       2. The highest risk for MOH is opioids, butalbital-containing combination analgesics, and acetaminophen-aspirin-caffeine combinations ^10^.
       3. Conflicting data for NSAIDs, some studies range risk from low in most studies to high in others.
2. How does aspirin differ from other NSAIDs?
   - Aspirin differs from NSAIDs as it irreversibly inhibits COX-1 & COX-2 decreasing thromboxane A2 (TXA2_)_ & prostaglandin formation.
   - It also has an antiplatelet effect that lasts 7 days.
3. How could the medications be administered differently to increase their effectiveness?

- To increase effectiveness, you would need to optimize dosages and frequency with each product.

**Slide 8:**

- Ask the student groups to answer question 6.
- After ~2 minutes choose a group to answer question 6.

The headaches are interfering with her ability to work and take care of her family. She has two sisters, neither of whom suffer from migraines. Eddie’s medical history is unremarkable. A brief physical exam is normal and neurological exam is within normal limits. You make a diagnosis of migraine headache with aura.

*Question 6:*

1. Which of the following medications would be your plan of action to treat Eddie’s migraine?
2. Sumatriptan 50 mg tablet
3. Ergotamine/caffeine 1mg/100mg tablet
4. Naproxen 500 mg tablet
5. Propranolol 10 mg tablet

**Slide 9:**

- Use this slide to summarize the answer to question 6.
- The slide is animated so the facilitator can reveal the correct answer following a discussion with the students.

*Summary of question 6:*

6. Which of the following medications would be your plan of action to treat Eddie’s migraine?

1. **Sumatriptan 50 mg tablet – Correct answer, text is color red on mouse click**
2. Ergotamine/caffeine 1mg/100mg tablet
3. Naproxen 500 mg tablet
4. Propranolol 10 mg tablet

**Slide 10:**

- Ask the student groups to answer question 7.
- After ~2 minutes choose a group to answer question 7.

*Questions 7:*

7. What is the mechanism of action and the desired outcome of the triptan family?

**Slide 11:**

- Use this slide to summarize the answer to question 7.
- The slide is animated so the facilitator can reveal the correct answer following a discussion with the students. There is a summary figure on this slide as well.

*Summary of question 7:*

7. What is the mechanism of action and the desired outcome of the triptan family?

- - Triptans activate the 5-hydroxytrptamine (5-HT) receptors - 1B/1D/1F which are Gαi coupled
    - Activation leads to the inhibition of adenylyl cyclase, decreasing cAMP and Protein Kinase A (PKA)
  - Decreased PKA activity leads to decreased vasodilation (increased vasoconstriction)
  - Decreased PKA leads to a decreased dopamine effect
    - Migraineurs are hypersensitive to dopamine and presents as nausea/vomiting
  - Activation of the 5-HT_1B_ receptor causes:
    - Vasoconstriction of meningeal, dural, and cerebral vessels
    - Inhibition of the release of vasoactive peptides including calcitonin gene-related peptide (CGRP), Substance P and Neurokinin A. This ultimately results in decreased vasodilation and inflammation.
      - Calcitonin Gene-Related Peptide (CGRP) and Substance P: produce inflammation of pain-sensitive meninges and dilation of affected cranial blood vessels which generates headache.
      - Neurokinin
    - Newest class available for migraine prophylaxis: Calcitonin Gene-Related Peptide (CGRP) monoclonal antibodies galcenezumab and fremanezumab – bind to CGRP peptide blocking its binding to the receptor, erenumab – receptor antagonist – competes with the binding of CGRP to the receptor to inhibit receptor function
    - Sensation of pain is reduced through the inhibition of the trigeminal neurons

**Slide 12:**

- Ask the student groups to answer question 8.
- After ~2 minutes choose a group to answer question 8.

*Question 8:*

8. What factors will impact triptan product selection?

**Slide 13:**

- Use this slide to summarize the answer to question 8.
- The slide is animated so the facilitator can reveal the correct answer following a discussion with the students.

*Summary of question 8:*

1. What factors will impact triptan product selection?
   - Note: The parameters described below allow focused conversation on drug kinetics vs. clinical practice guidelines. Choice of agent needs to be individualized; pharmacologic properties and routes will help aid in selection.
   - Efficacy
     - At 2 hours - best performance:
       - Eletriptan > rizatriptan > zolmitriptan > sumatriptan > almotriptan > naratriptan > frovatriptan
     - At 24 hours – best performance:
       - Eletriptan > zolmitriptan > almotriptan > rizatriptan > sumatriptan
   - Formulation
     - Oral, ODT, Nasal, SubQ
   - Duration of action
     - Fast acting
     - Long acting

**Slide 14:**

- Ask the student groups to answer question 9.
- After ~2 minutes choose a group to answer question 9

*Question 9:*

9. What side effects and safety concerns are associated with triptan use?

**Slide 15:**

- Use this slide to summarize answer to question 9.
- The slide is animated so the facilitator can reveal the correct answer following a discussion with the students.

*Summary of question 9:*

9. What side effects and safety concerns are associated with triptan use?

- Side effects include: chest tightness, altered sensation (tingling/warmth/burning), dizziness, nausea/vomiting, dry mouth, muscle weakness, fatigue, neck pain, somnolence, injection site reactions
- Triptans are contraindicated for individuals with the following conditions:
  - - Ischemic heart disease
    - Uncontrolled hypertension, the concern is due to the impact of the 5-HT_1B_ receptors on vascular smooth muscle
    - Individuals within 24h of ergot – concern is due to serotonin syndrome
    - MAO inhibitor – concern is due to serotonin syndrome
- Eletriptan
  - - Avoid use in combination with CYP3A4 inhibitors
- For more information see ^11^ ^12^.

**Slide 16:**

- Ask the student groups to answer question 10.
- After ~2 minutes choose a group to answer question 10.

*Question 10:*

10. It is revealed that the patient is taking an estrogen/progesterone contraception. What is the association between contraceptive medications and migraine headaches?

**Slide 17:**

- Use this slide to summarize the answer to question 10.
- The slide is animated so the facilitator can reveal the correct answer following a discussion with the students.

*Summary of question 10:*

10. It is revealed that the patient is taking an estrogen/progesterone contraception. What is the association between contraceptive medications and migraine headaches?

- Decline in estrogen can be a migraine trigger in women ^13^.
  - At the beginning of menstrual cycle, there is a natural decline in endogenous estrogen.
  - The hormone-free interval of oral contraceptives can cause/contribute to headaches.
  - Alternative options are to consider different hormonal contraceptive:
    - Examples are extended cycle oral contraceptive regimen or oral contraceptive with shorted pill free interval (4 days instead of 7).
- Ischemic stroke risk in women taking oral contraceptives:
- Increased risk of ischemic stroke in individuals using combined oral contraceptives with additional stroke risk factors (e.g. smoking, hypertension, and migraine with aura)
  - No specific guidelines exist for OCP prescribing for patients with migraine with aura.
- Use of non-estrogen contraception, (Progestogen intrauterine device, intramuscular, implants, pills), is recommended for women with multiple risk factors for stroke.
- The American College of Obstetricians and Gynecologists guidelines recommend progestin-only (pill, IUD, injection, and implant) or nonhormonal (copper IUD, surgery, and barrier) contraceptive methods for women with migraine with focal neurological signs (for example, hemiplegic migraine), women ≥35 years of age, and smokers ^14^.

**Slide 18:**

- Ask the student groups to answer the question 11a-d.
- After ~6 minutes choose a group to answer question 11a-d.
  - Note: there are four parts to this question but they should not take as long as 4 full questions.

*Question 11 a-d:*

11. Does your treatment change if the patient is:

a) Hypertensive BP 130/85, being controlled with lisinopril?

b) Has uncontrolled hypertension?

c) Experiencing migraines greater than 4 hours in duration after prescribed treatment?

d) Vomiting at migraine onset?

**Slide 19 - 22:**

- Use these slides to summarize the answers to questions 11a-d.
- Slide 19 is animated so the facilitator can reveal the correct answer following a discussion with the students.

*Summary of questions 11a-d (slide is animated so each scenario can be discussed separately):*

11. Does your treatment change if the patient is:

a) Hypertensive BP 130/85, being controlled with lisinopril or b) has uncontrolled hypertension?

- - There is no drug: drug interaction to be concerned with if the patient if being treated with lisinopril. Lisinopril and triptans have no adverse reactions.
  - Caution should be used if the patient is using NSAIDs with an ACE inhibitor
  - Individuals with uncontrolled hypertension should have their hypertension addressed adding additional medications
    - The treatment of hypertension will reduce the prevalence of headache

c) Experiencing migraines greater than 4 hours in duration after prescribed treatment?

- - The best recommendation would be to change the prescribed product to one with a longer duration of action.
  - You could additionally add an NSAID to the treatment regime.

d) Vomiting at migraine onset?

- - For this presentation it is best to change the formulation of the drug. Orally disintegrating tablets, SubQ or nasal would be better formulations for drug administration.
  - In addition, the individual could be prescribed and antiemetic to alleviate vomiting.
  - Alternative options would include a dopamine antagonist such as:
    - Metoclopramide, this drug also blocks 5-HT in chemoreceptor trigger zone at high doses.
      - This enhances acetylcholine in upper GI, enhancing gastric emptying
        - Gastric emptying is reduced during migraine
    - Prochlorperazine or chlorpromazine
      - Combine with diphenhydramine to decrease akathisia (restlessness) and dystonic (rhythmic) reactions
  - Migraine treatment options (*Summarized in slide 20 -22*)^15^:
    - DHE nasal spray (Or IV if in an emergency department or hospital based setting) – α-blocker with 5-HT-1B/1D agonist activity; used for intractable migraine (>72hr)
    - Ergotamine/caffeine – effective as sumatriptan, but has more side effects; ergotamine alone is less efficacious for migraine relief
    - IV dexamethasone reduces rate of early headache recurrence
  - Opioids would be a last resort as they are less effective and can lead to medication overuse and/or addiction.

**Slide 23:**

- Ask the student groups to answer the question 12.
- After ~2 minutes choose a group to answer question 12.

*Question 12:*

For the acute treatment of her migraine headaches, you prescribe sumatriptan 50 mg tablet with directions to take 1 tablet at onset of migraine, may repeat in 2 hours if not resolved. At her 2-month follow up appointment you inquire if this therapy has been effective. The patient states that the medication has helped the pain and duration of her headache, but the frequency has not changed. She is having to take the rescue medication 3 or more times per week.

12. What alternatives could you try now?

**Slide 24:**

- Use this slide to summarize the answer to question 12.

*Summary of question 12:*

12. What alternatives could you try now?

- Indications for use of prophylactic therapy to help prevent migraines:
  - 2 or more attacks per month that produce disability lasting 3 or more days per month
  - Failure or contraindication of acute treatment
  - If the patient is using medication for acute treatment greater than 2 times a week.
  - If there is presence of uncommon migraine conditions including but not limited to: hemiplegic migraine, migraine with prolonged aura.
- Goals of prophylactic therapy:
  - To reduce attack frequency, severity and duration
  - To improve responsiveness of treatment of acute attacks
  - To reduce the disability to the individual
- Options:
  - Tricyclic antidepressants (TCA) block serotonin transporters (SERT) and norepinephrine transporters (NET), low dose daily at bedtime/evening
    - Selective Serotonin Reuptake Inhibitor (SSRI) or Serotonin and Norepinephrine Reuptake Inhibitors (SNRI) are found to be less efficacious for migraine prevention
  - Antiepileptics with the overall effect being to decrease repetitive neuronal firing
    - Valproate: Induces a Na^+^ & Ca^2++^ blockade and increases GABA production
    - Topiramate: Blocks Na^+^ channels and increases GABA production
  - Antihypertensives:
    - Propranolol – β-blocker (Gαs)
    - Calcium channel blocker
    - ACE/ARB
    - Diuretics
  - Calcitonin Gene-Related Peptide (CGRP) antagonists
    - erenumab, fremanezumab, galcanezumab

**Slide 25:**

Summary:

Different types of headaches:

- Tension: Prophylaxis – TCA; Acute Treatment: acetaminophen, NSAIDs
- Cluster: Prophylaxis – verapamil, melatonin, suboccipital injection of betamethasone, lithium; Treatment – triptans (nasal/SubQ), oxygen, intranasal lidocaine
- Treatment should be progressive to ensure patient relief and improved quality of life.
- Botox is FDA approved for prophylaxis in chronic headaches.
  - This may present as a conversation topic and it is important to note it does not currently have FDA indication for Tension-type headache, only chronic migraine. It leads to a small reduction in the number of headaches per month, but results are not statistically significant. For more information see ^16^.

**Slide 26 -27:**

References:

1. Pelletier-Dattu C. *Lange Smart Charts: Pharmacology, 2nd Edition* McGraw-Hill Companies 2015.

2. Bajwa Z, Smith J. Acute treatment of migraine in adults. UpToDate Web site. Published 2018. Accessed2018.

3. Sinclair AJ, Sturrock A, Davies B, Matharu M. Headache management: pharmacological approaches. *Pract Neurol.* 2015;15(6):411-423.

4. Clarkson C. Physiology Concepts & Clinical Context – Migraine Headaches. Tulane University. Published 2016. AccessedMarch 27, 2018.

5. Bajwa Z, Smith J. Preventative treatment of migraine in adults. Accessed 2018, 2018.

6. Grosser T, Smyth E, FitzGerald G. Pharmacotherapy of Inflammation, Fever, Pain, and Gout. In: Brunton L, Hilal-Dandan R, Knollmann B, eds. *Goodman & Gilman's: The Pharmacological Basis of Therapeutics.* 13e ed. New York, NY: McGraw Hill.

7. Lipton RB, Diener HC, Robbins MS, Garas SY, Patel K. Caffeine in the management of patients with headache. *J Headache Pain.* 2017;18(1):107.

8. Bigal ME, Lipton RB. Excessive acute migraine medication use and migraine progression. *Neurology.* 2008;71(22):1821-1828.

9. Oleson J. Headache Classification Committee of the International Headache Society (IHS) The International Classification of Headache Disorders, 3rd edition. *Cephalalgia.* 2018;38(1):1-211.

10. Thorlund K, Sun-Edelstein C, Druyts E, et al. Risk of medication overuse headache across classes of treatments for acute migraine. *J Headache Pain.* 2016;17(1):107.

11. Sibley D, Hazelwood L, Amara S. 5-Hydroxytryptamine (Serotonin) and Dopamine. In: Brunton L, R H-D, Knollmann B, eds. *Goodman & Gilman's: The Pharmacological Basis of Therapeutics.* New York, NY: McGraw Hill.

12. Katzung B. Serotonin, & the Ergot Alkaloids. In: Katzung B, ed. *Basic & Clinical Pharmacology.* 14e ed. New York, NY: McGraw Hill.

13. Sheikh HU, Pavlovic J, Loder E, Burch R. Risk of Stroke Associated With Use of Estrogen Containing Contraceptives in Women With Migraine: A Systematic Review. *Headache.* 2018;58(1):5-21.

14. Carlton C, Banks M, Sundararajan S. Oral Contraceptives and Ischemic Stroke Risk. *Stroke.* 2018;49(4):e157-e159.

15. Loder E, Burch R, Rizzoli P. The 2012 AHS/AAN guidelines for prevention of episodic migraine: a summary and comparison with other recent clinical practice guidelines. *Headache.* 2012;52(6):930-945.

16. Jackson JL, Kuriyama A, Hayashino Y. Botulinum Toxin A for Prophylactic Treatment of Migraine and Tension Headaches in Adults. *Jama.* 2012;307(16):1736-1745.
