# Supplementary material for: An Interactive Process for Delivering Pharmacologic Interventions for Migraine Headache to First-Year Medical Students
Source: MedEdPORTAL. 2020 Feb 7;16:10877. doi: 10.15766/mep_2374-8265.10877 (PMC7012313; doi:10.15766/mep_2374-8265.10877)
Supplement: Supplementary file 1 — A. Migraine Facilitator Guide.docx B. Advance Preparation Materials.docx C. Student Migraine Presentation.pptx D. Facilitator Migraine Presentation.pptx [file mep-16-10877-s001.zip › B. Advance Preparation Materials.docx]

Appendix B: Advance preparation materials for students

Prior to coming to class use the table referenced below to address the following learning objectives and come prepared to discuss.

- - Identify classes of medications utilized for migraines.
  - List agents within each class and recognized drug name endings associated with these agents.

Advanced preparation material:

Review the **Lange Smart Charts: Pharmacology 2^nd^ Edition, Section VI.: Drugs for the treatment of migraines; Table titled: Classification of drugs used for the treatment of migraines, pg 171.**^1^

**References:**

1. Pelletier-Dattu CE. *Lange Smart Charts: Pharmacology, 2nd Edition*. 2nd ed. (Pelletier-Dattu CE, ed.). McGraw-Hill Companies; 2015.
