# Supplementary material for: An Interactive Process for Delivering Pharmacologic Interventions for Migraine Headache to First-Year Medical Students
Source: MedEdPORTAL. 2020 Feb 7;16:10877. doi: 10.15766/mep_2374-8265.10877 (PMC7012313; doi:10.15766/mep_2374-8265.10877)
Supplement: Supplementary file 1 — A. Migraine Facilitator Guide.docx B. Advance Preparation Materials.docx C. Student Migraine Presentation.pptx D. Facilitator Migraine Presentation.pptx [file mep-16-10877-s001.zip › C. Student Migraine Presentation.pptx]

## Slide 1
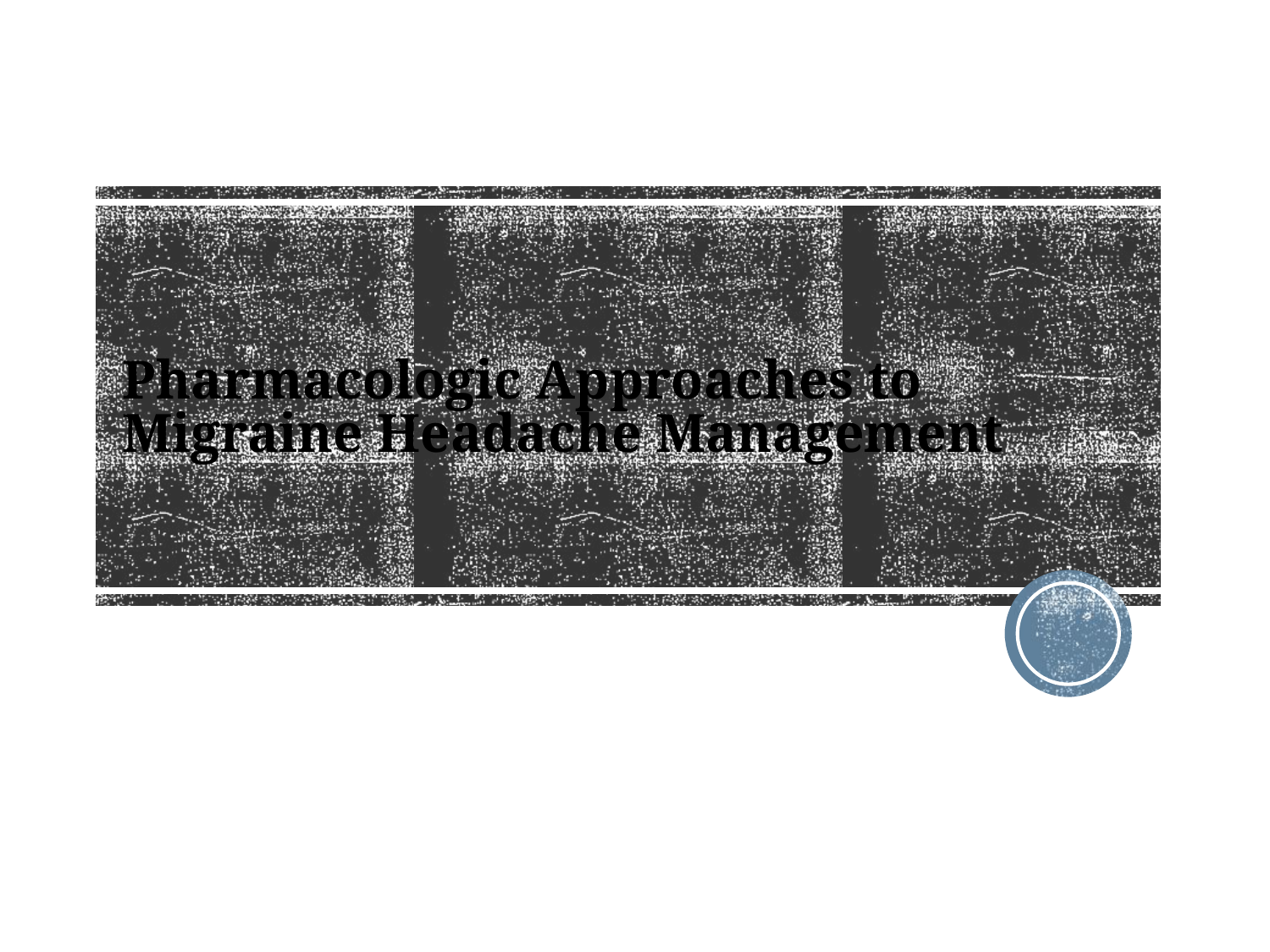

# Pharmacologic Approaches to Migraine Headache Management

## Slide 2
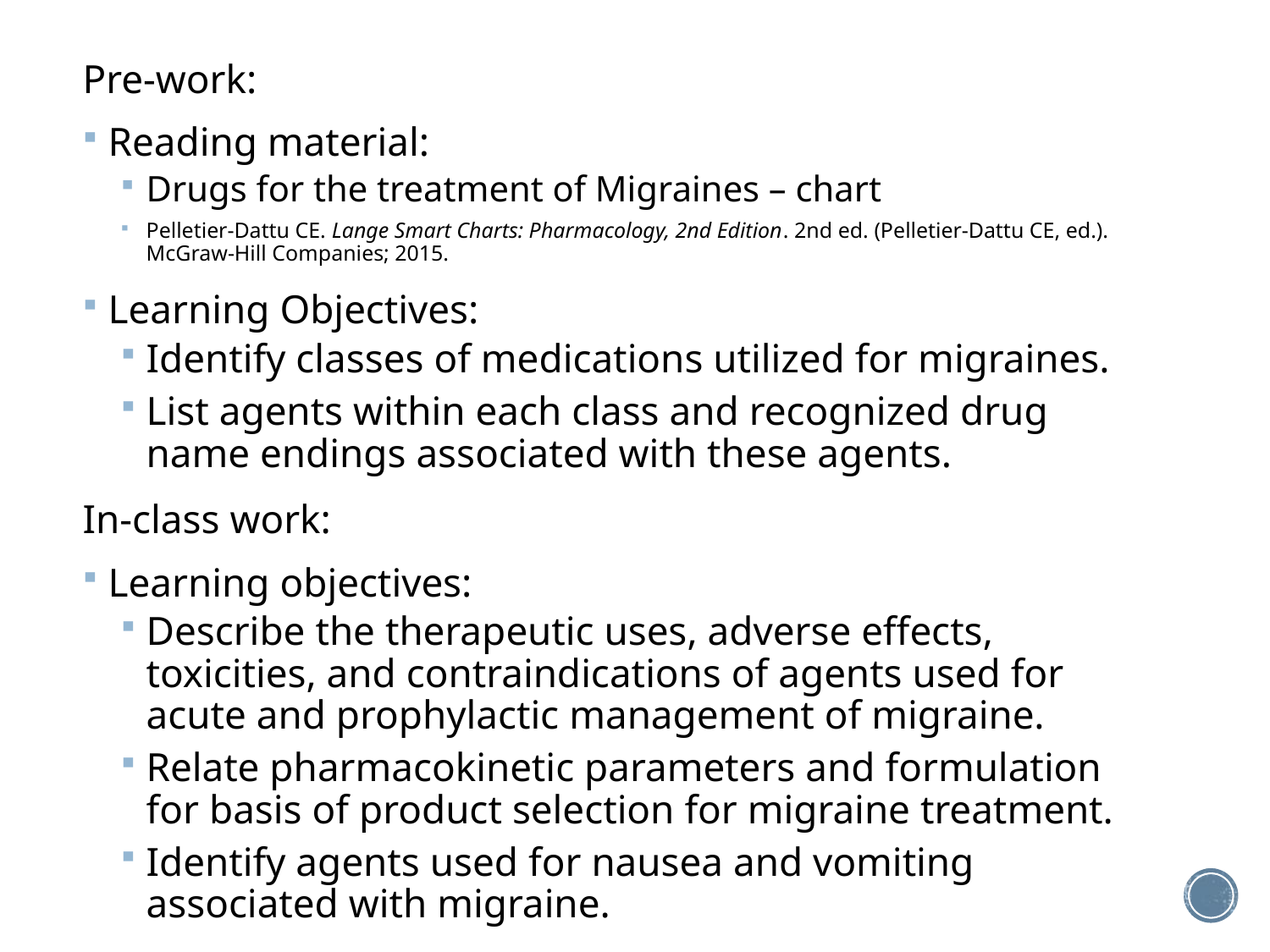

Pre-work:
Reading material:
Drugs for the treatment of Migraines – chart
Pelletier-Dattu CE. Lange Smart Charts: Pharmacology, 2nd Edition. 2nd ed. (Pelletier-Dattu CE, ed.). McGraw-Hill Companies; 2015.
Learning Objectives:
Identify classes of medications utilized for migraines.
List agents within each class and recognized drug name endings associated with these agents.
In-class work:
Learning objectives:
Describe the therapeutic uses, adverse effects, toxicities, and contraindications of agents used for acute and prophylactic management of migraine.
Relate pharmacokinetic parameters and formulation for basis of product selection for migraine treatment.
Identify agents used for nausea and vomiting associated with migraine.

## Slide 3
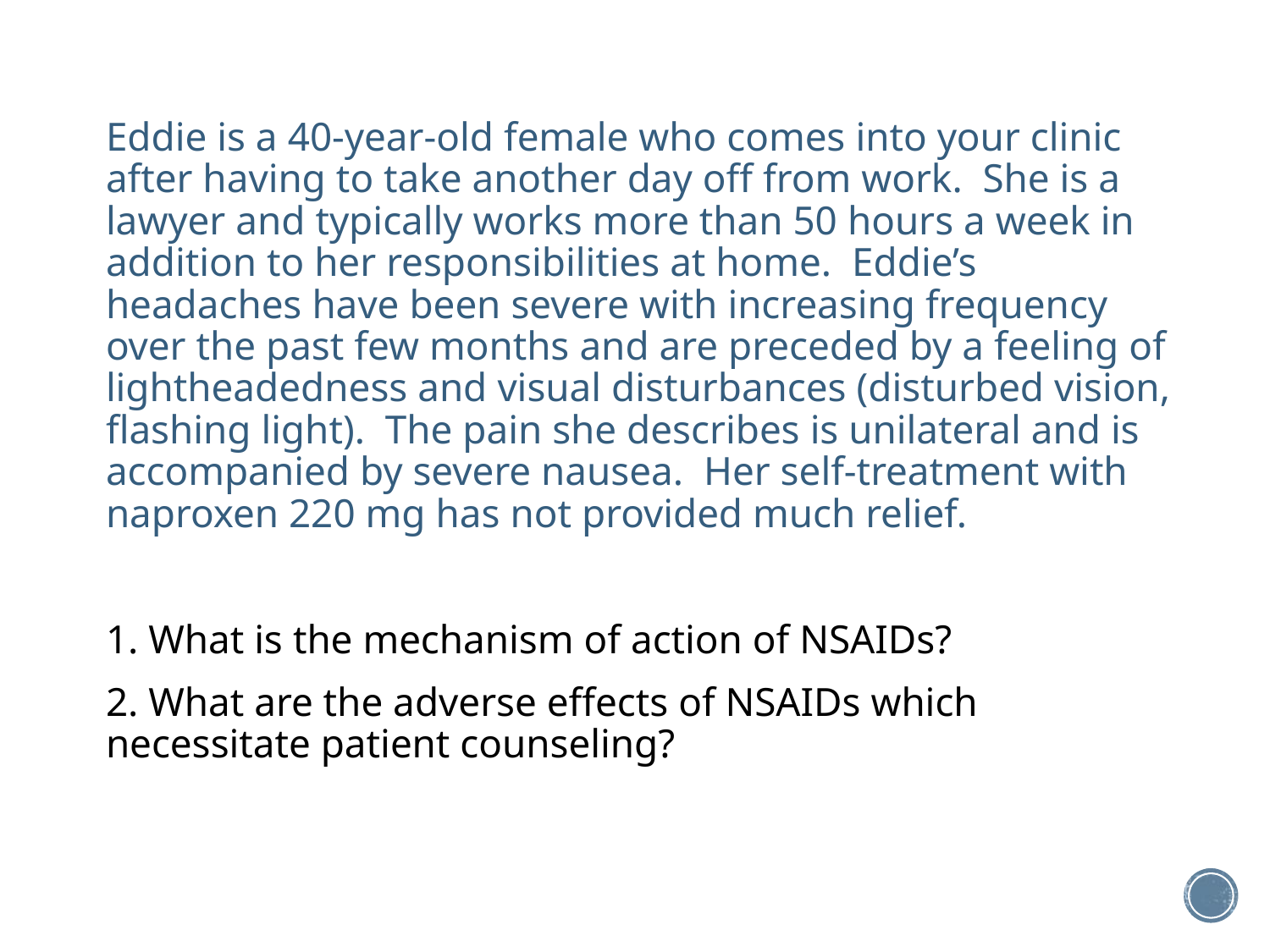

Eddie is a 40-year-old female who comes into your clinic after having to take another day off from work. She is a lawyer and typically works more than 50 hours a week in addition to her responsibilities at home. Eddie’s headaches have been severe with increasing frequency over the past few months and are preceded by a feeling of lightheadedness and visual disturbances (disturbed vision, flashing light). The pain she describes is unilateral and is accompanied by severe nausea. Her self-treatment with naproxen 220 mg has not provided much relief.
1. What is the mechanism of action of NSAIDs?
2. What are the adverse effects of NSAIDs which necessitate patient counseling?

## Slide 4
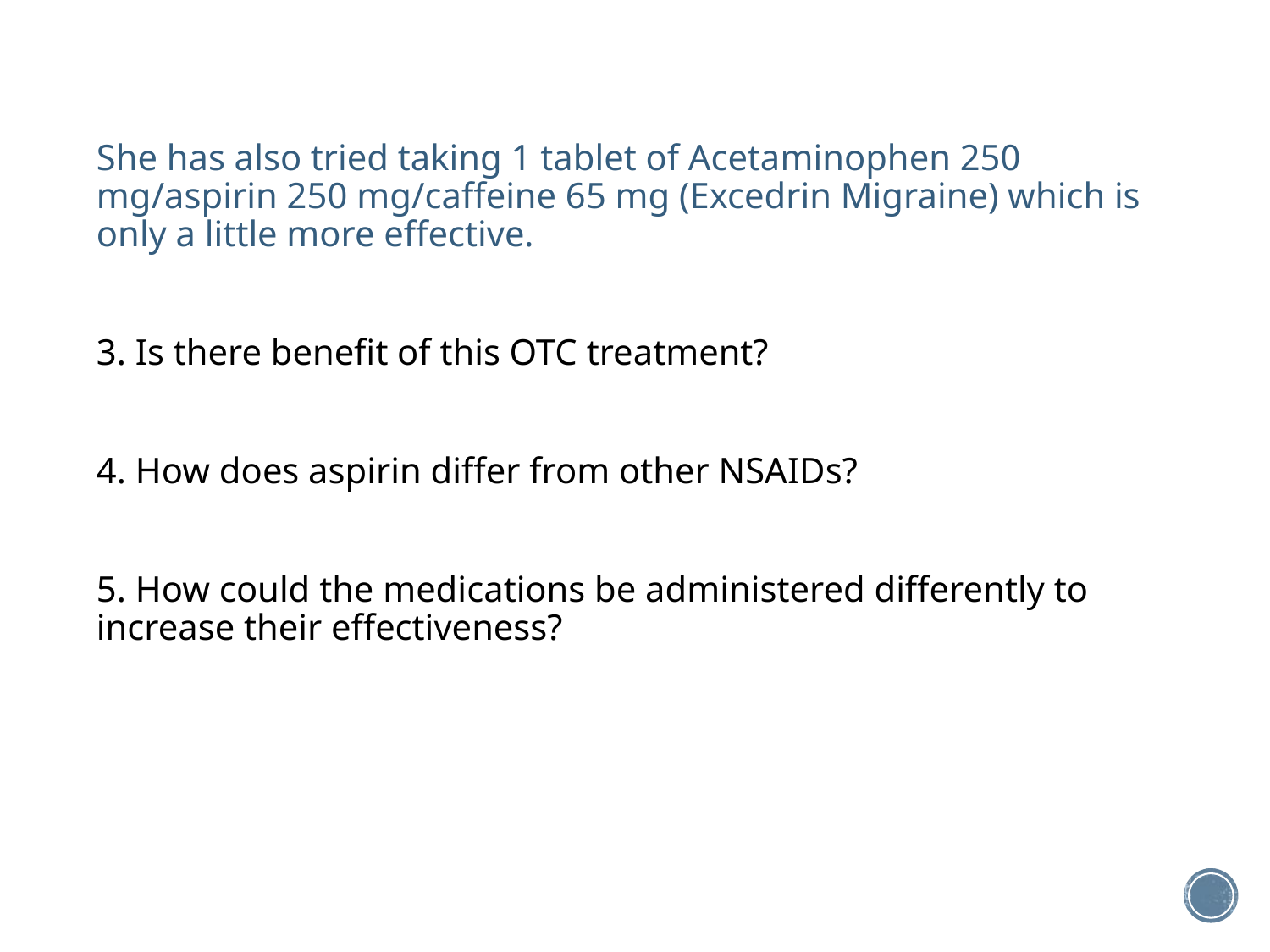

She has also tried taking 1 tablet of Acetaminophen 250 mg/aspirin 250 mg/caffeine 65 mg (Excedrin Migraine) which is only a little more effective.
3. Is there benefit of this OTC treatment?
4. How does aspirin differ from other NSAIDs?
5. How could the medications be administered differently to increase their effectiveness?

## Slide 5
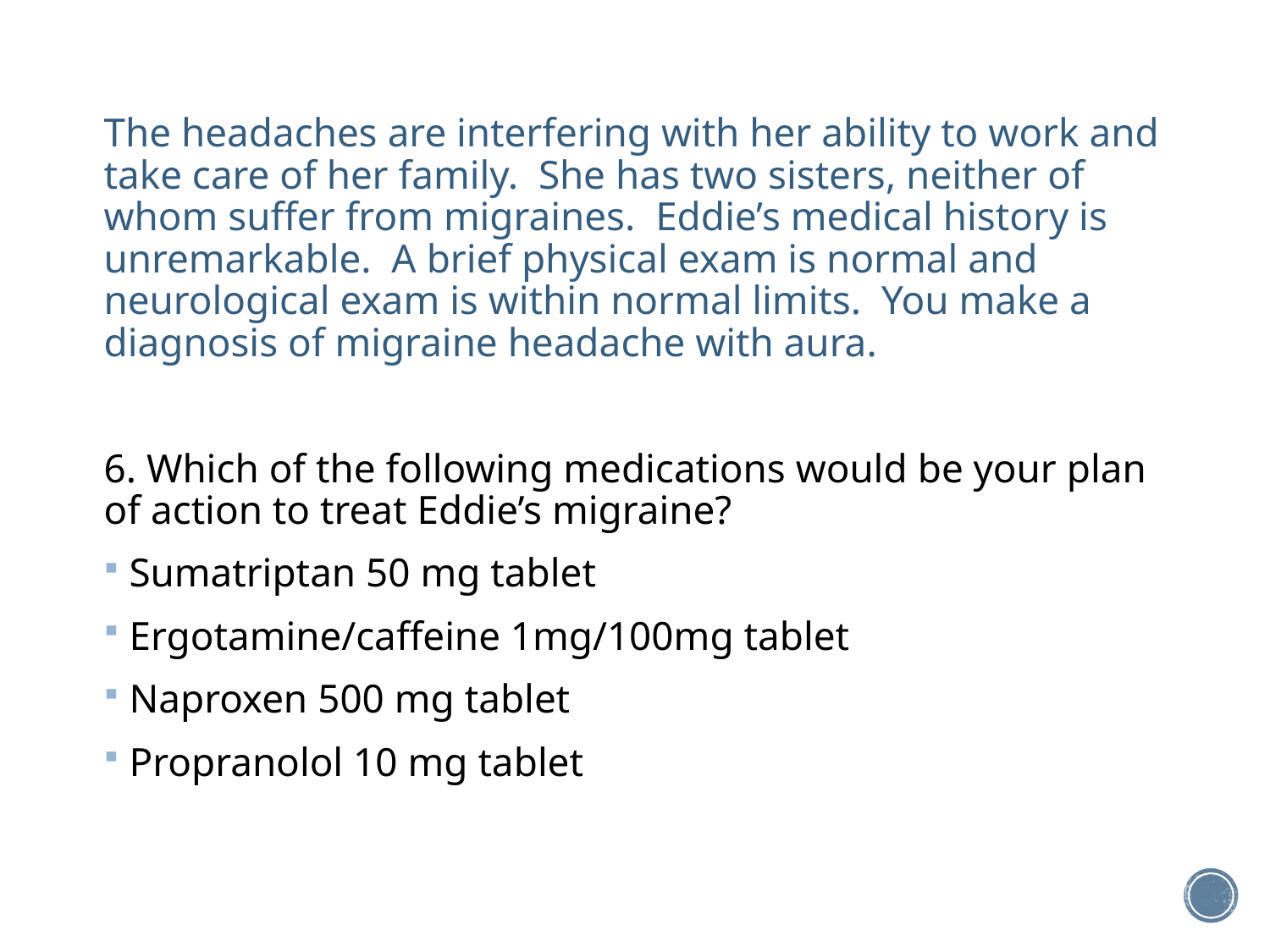

The headaches are interfering with her ability to work and take care of her family. She has two sisters, neither of whom suffer from migraines. Eddie’s medical history is unremarkable. A brief physical exam is normal and neurological exam is within normal limits. You make a diagnosis of migraine headache with aura.
6. Which of the following medications would be your plan of action to treat Eddie’s migraine?
Sumatriptan 50 mg tablet
Ergotamine/caffeine 1mg/100mg tablet
Naproxen 500 mg tablet
Propranolol 10 mg tablet

## Slide 6
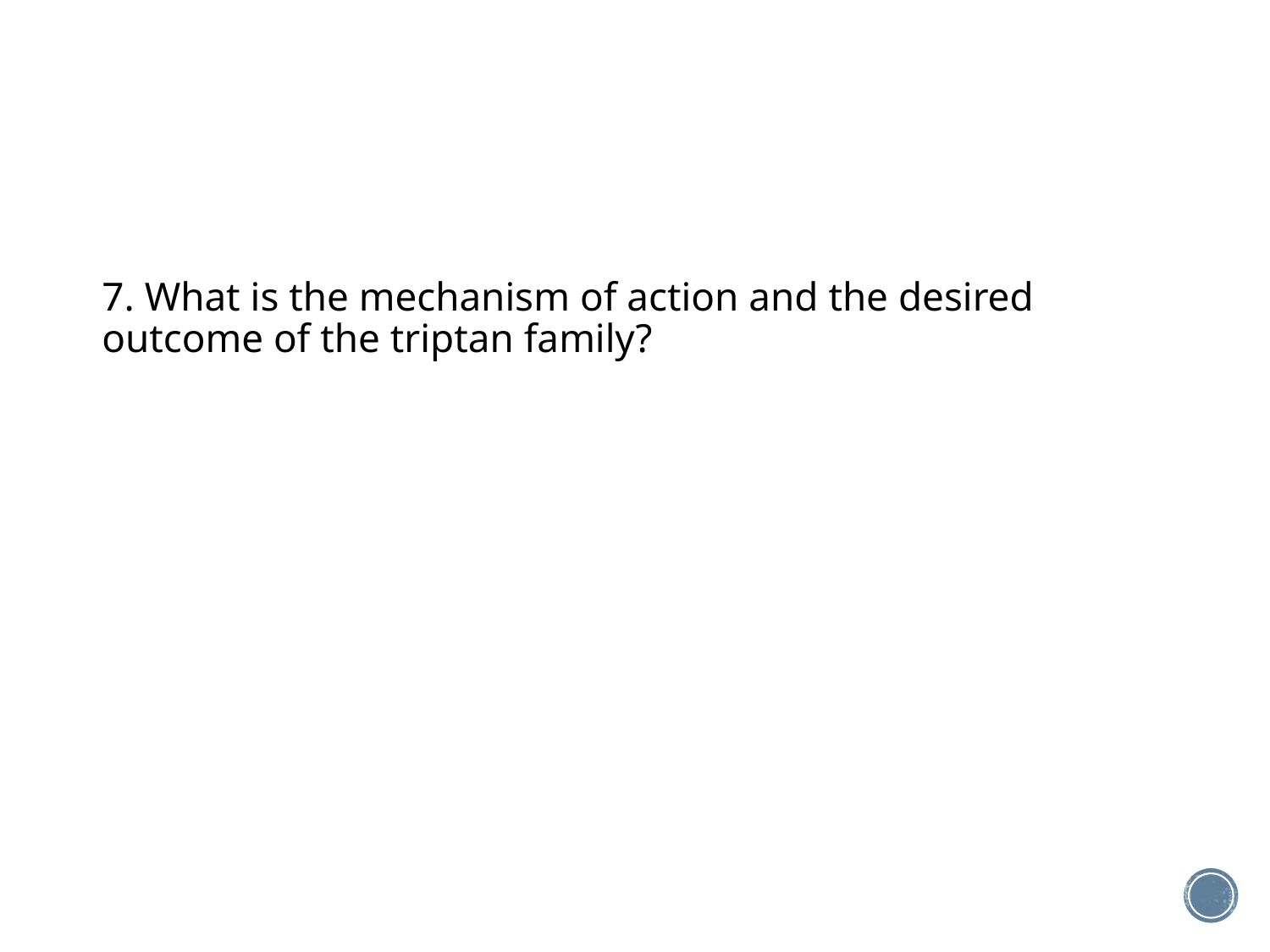

7. What is the mechanism of action and the desired outcome of the triptan family?

## Slide 7
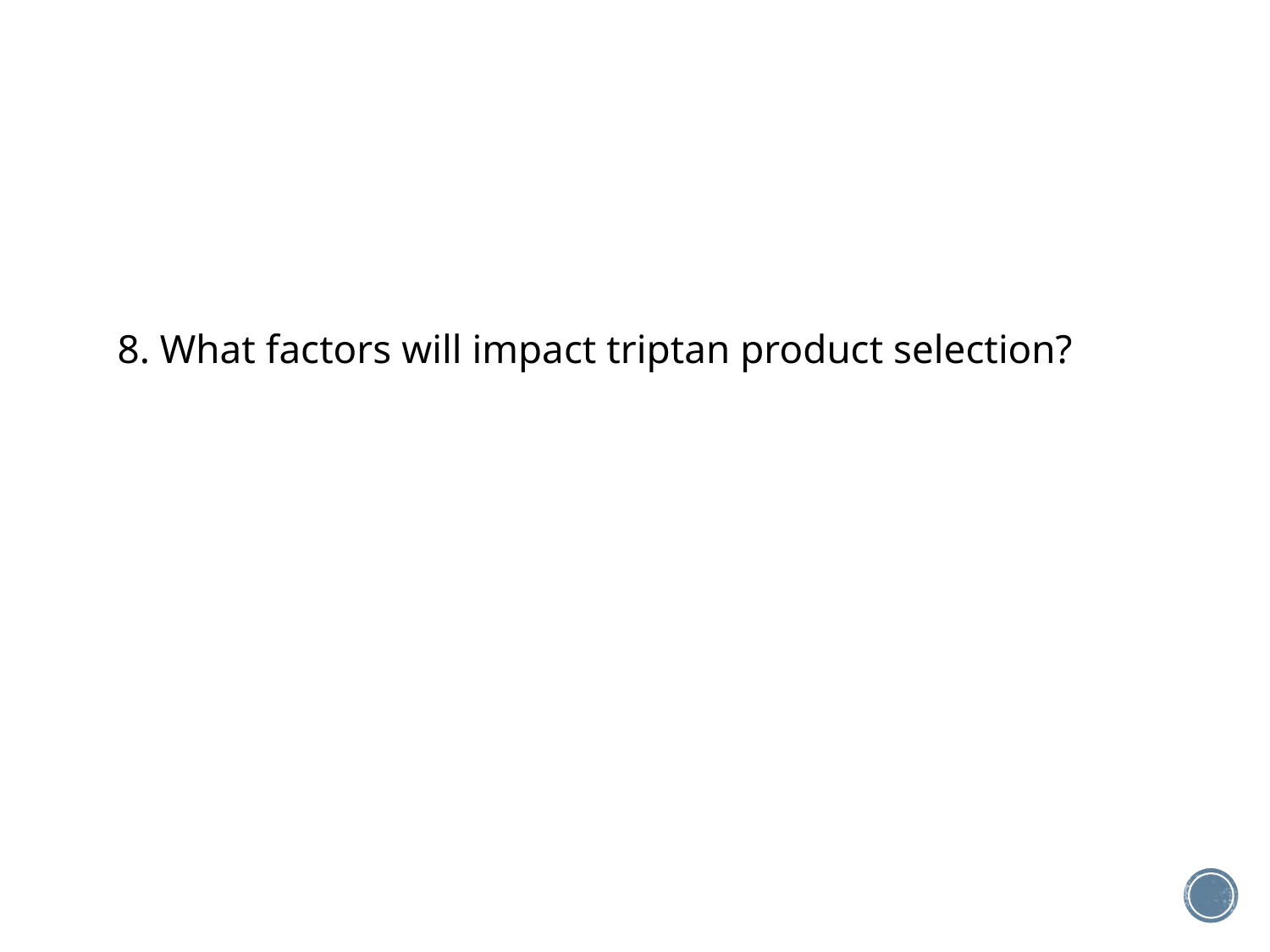

8. What factors will impact triptan product selection?

## Slide 8
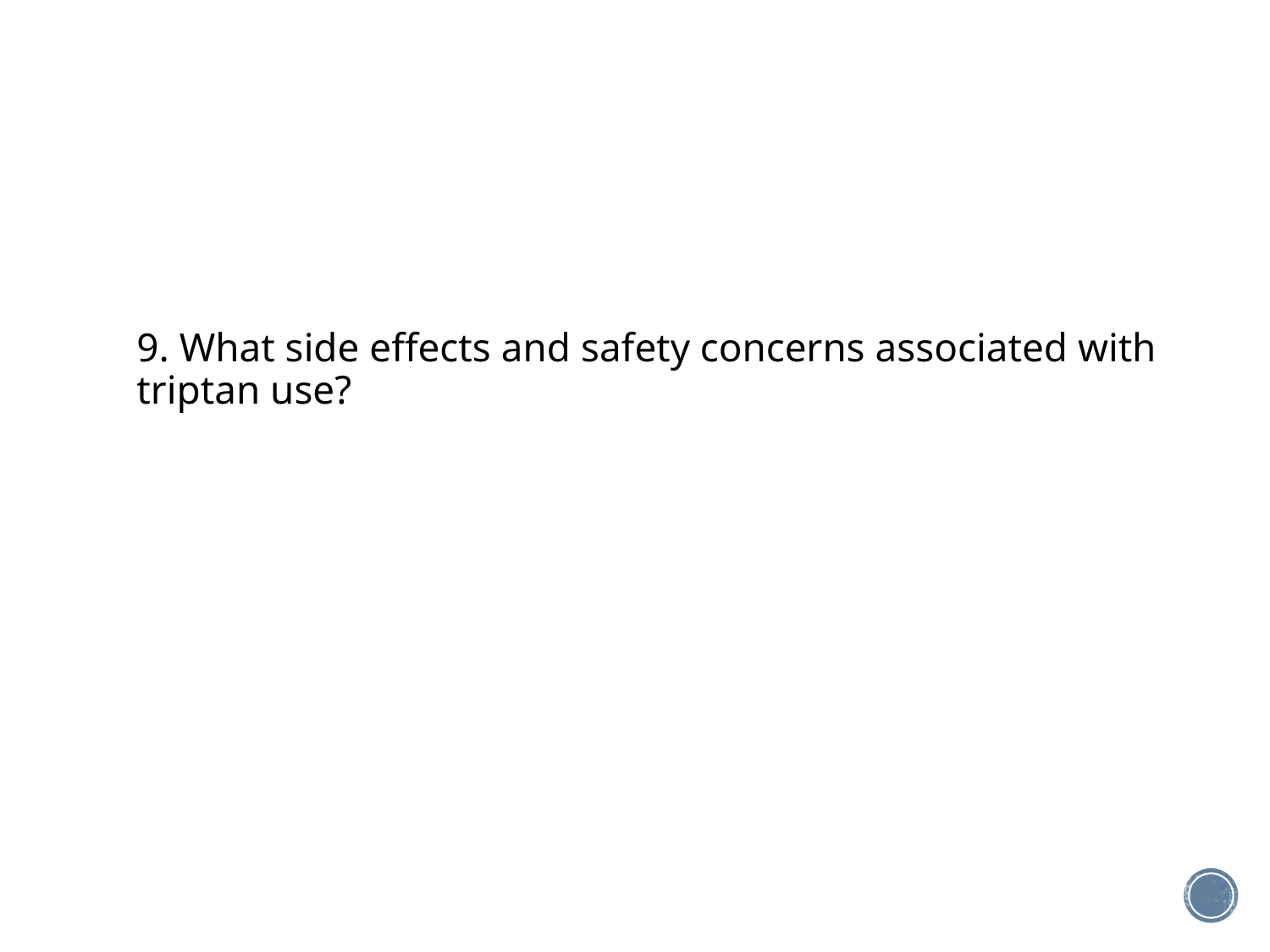

9. What side effects and safety concerns associated with triptan use?

## Slide 9
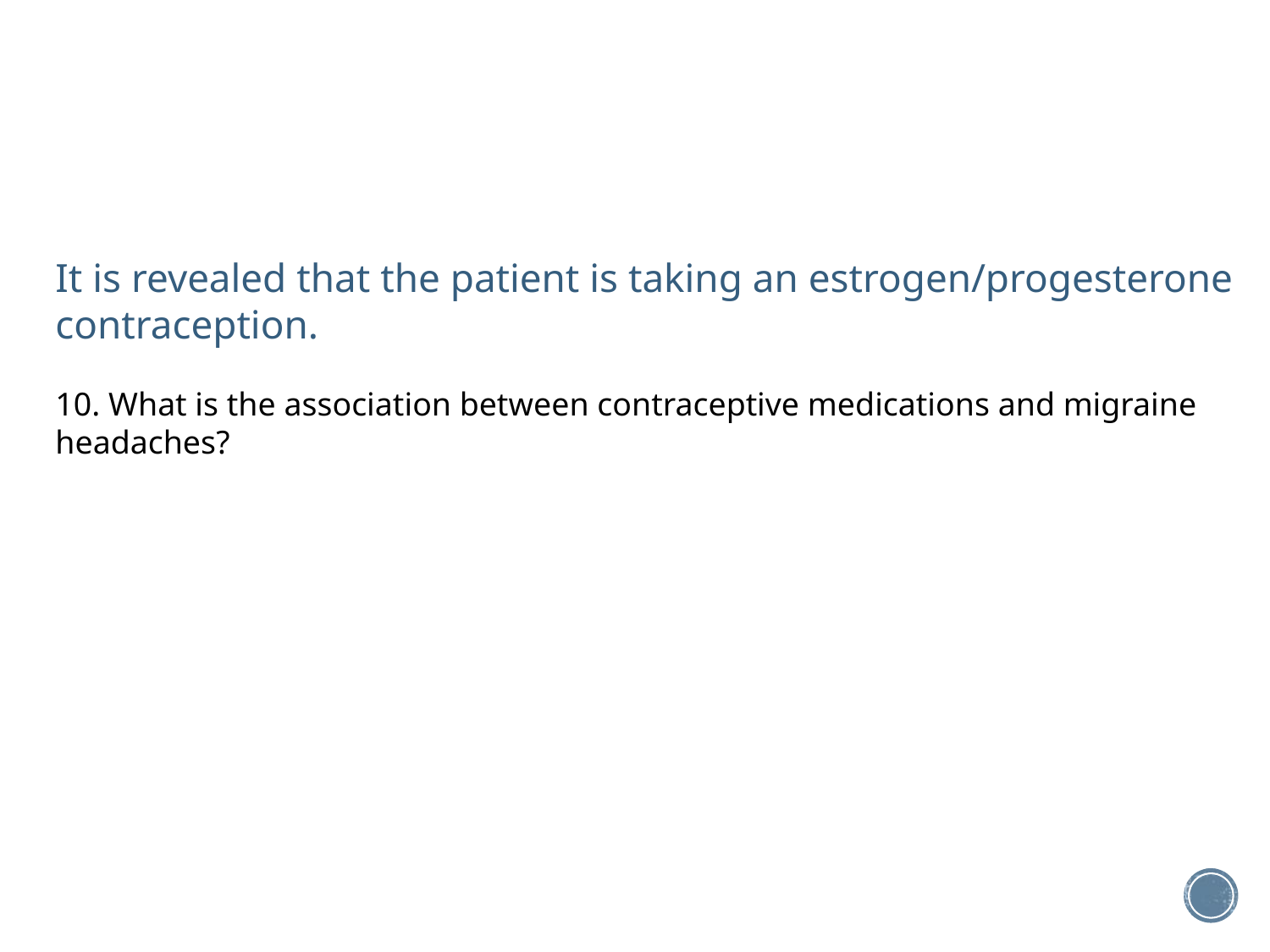

It is revealed that the patient is taking an estrogen/progesterone contraception.
10. What is the association between contraceptive medications and migraine headaches?

## Slide 10
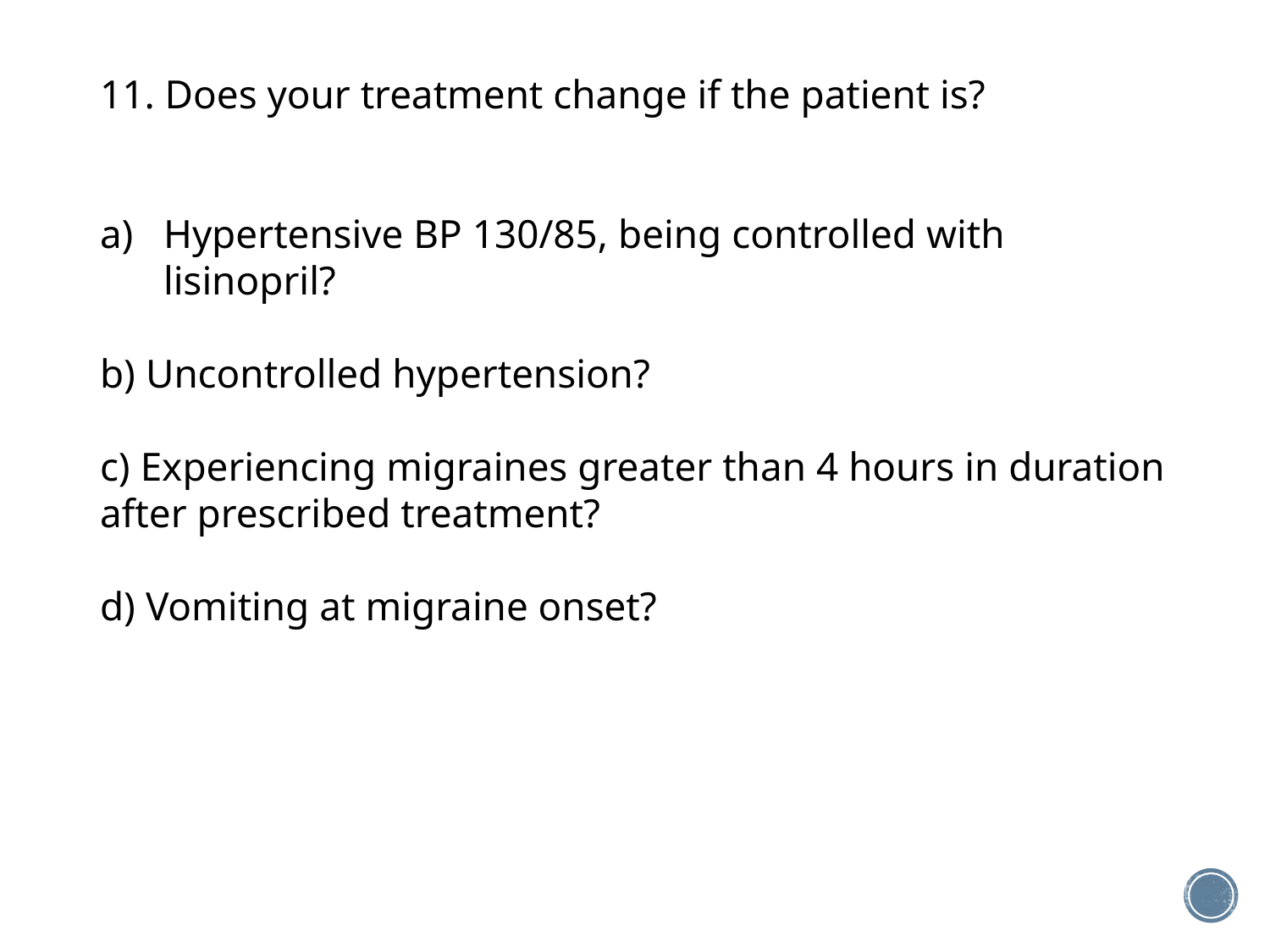

11. Does your treatment change if the patient is?
Hypertensive BP 130/85, being controlled with lisinopril?
b) Uncontrolled hypertension?
c) Experiencing migraines greater than 4 hours in duration after prescribed treatment?
d) Vomiting at migraine onset?

## Slide 11
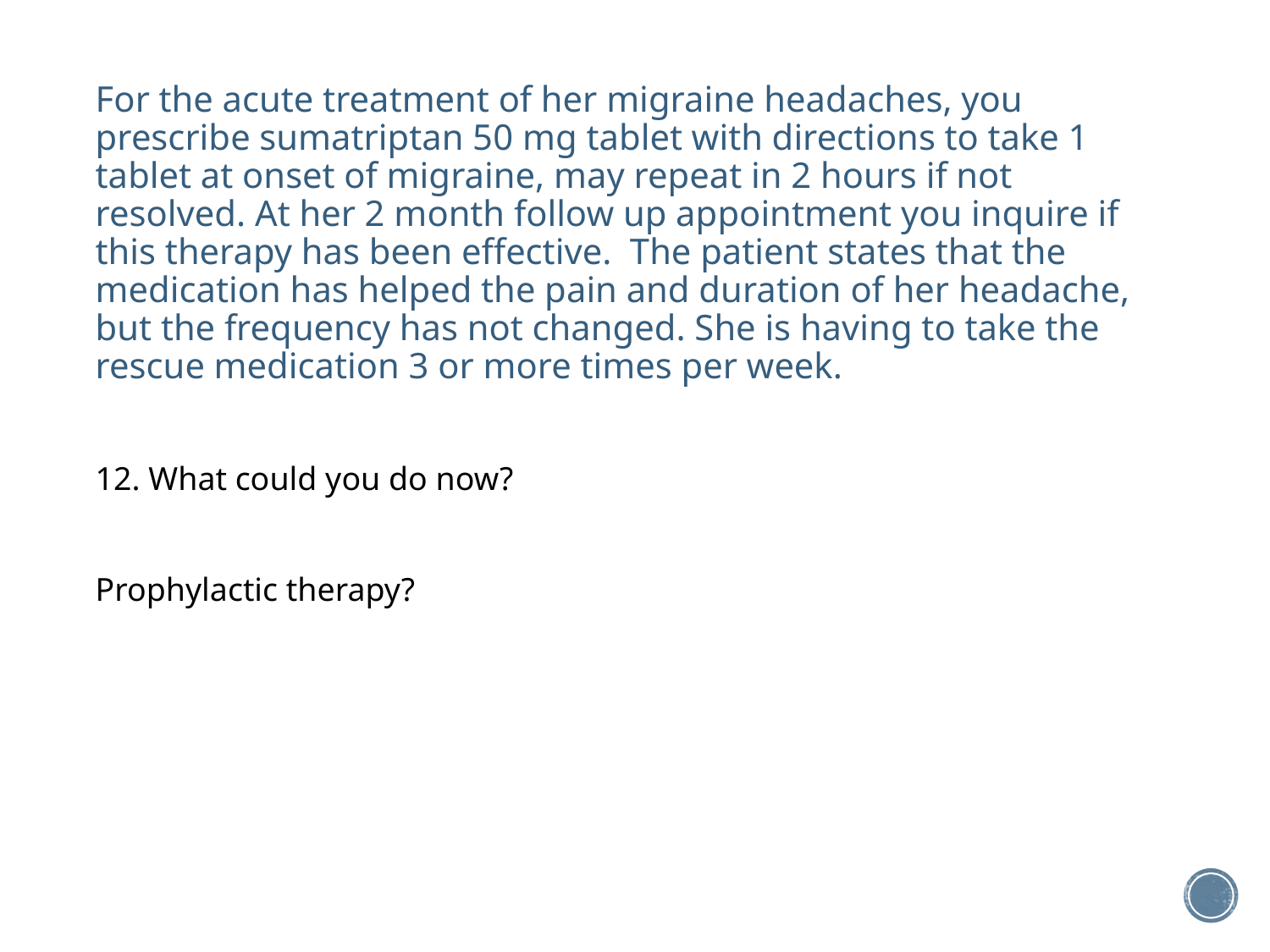

For the acute treatment of her migraine headaches, you prescribe sumatriptan 50 mg tablet with directions to take 1 tablet at onset of migraine, may repeat in 2 hours if not resolved. At her 2 month follow up appointment you inquire if this therapy has been effective. The patient states that the medication has helped the pain and duration of her headache, but the frequency has not changed. She is having to take the rescue medication 3 or more times per week.
12. What could you do now?
Prophylactic therapy?

## Slide 12
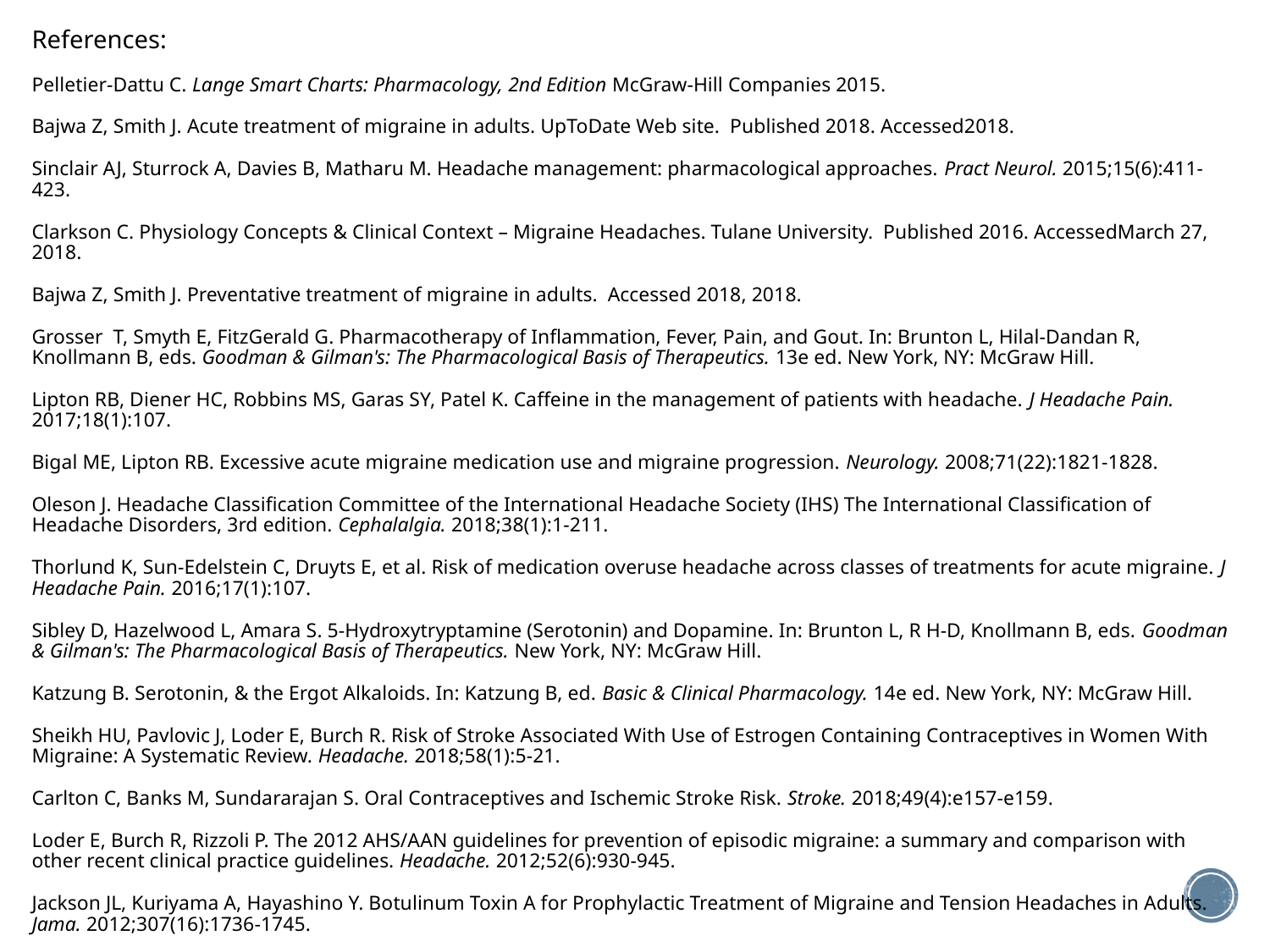

References:
Pelletier-Dattu C. Lange Smart Charts: Pharmacology, 2nd Edition McGraw-Hill Companies 2015.
Bajwa Z, Smith J. Acute treatment of migraine in adults. UpToDate Web site. Published 2018. Accessed2018.
Sinclair AJ, Sturrock A, Davies B, Matharu M. Headache management: pharmacological approaches. Pract Neurol. 2015;15(6):411-423.
Clarkson C. Physiology Concepts & Clinical Context – Migraine Headaches. Tulane University. Published 2016. AccessedMarch 27, 2018.
Bajwa Z, Smith J. Preventative treatment of migraine in adults. Accessed 2018, 2018.
Grosser T, Smyth E, FitzGerald G. Pharmacotherapy of Inflammation, Fever, Pain, and Gout. In: Brunton L, Hilal-Dandan R, Knollmann B, eds. Goodman & Gilman's: The Pharmacological Basis of Therapeutics. 13e ed. New York, NY: McGraw Hill.
Lipton RB, Diener HC, Robbins MS, Garas SY, Patel K. Caffeine in the management of patients with headache. J Headache Pain. 2017;18(1):107.
Bigal ME, Lipton RB. Excessive acute migraine medication use and migraine progression. Neurology. 2008;71(22):1821-1828.
Oleson J. Headache Classification Committee of the International Headache Society (IHS) The International Classification of Headache Disorders, 3rd edition. Cephalalgia. 2018;38(1):1-211.
Thorlund K, Sun-Edelstein C, Druyts E, et al. Risk of medication overuse headache across classes of treatments for acute migraine. J Headache Pain. 2016;17(1):107.
Sibley D, Hazelwood L, Amara S. 5-Hydroxytryptamine (Serotonin) and Dopamine. In: Brunton L, R H-D, Knollmann B, eds. Goodman & Gilman's: The Pharmacological Basis of Therapeutics. New York, NY: McGraw Hill.
Katzung B. Serotonin, & the Ergot Alkaloids. In: Katzung B, ed. Basic & Clinical Pharmacology. 14e ed. New York, NY: McGraw Hill.
Sheikh HU, Pavlovic J, Loder E, Burch R. Risk of Stroke Associated With Use of Estrogen Containing Contraceptives in Women With Migraine: A Systematic Review. Headache. 2018;58(1):5-21.
Carlton C, Banks M, Sundararajan S. Oral Contraceptives and Ischemic Stroke Risk. Stroke. 2018;49(4):e157-e159.
Loder E, Burch R, Rizzoli P. The 2012 AHS/AAN guidelines for prevention of episodic migraine: a summary and comparison with other recent clinical practice guidelines. Headache. 2012;52(6):930-945.
Jackson JL, Kuriyama A, Hayashino Y. Botulinum Toxin A for Prophylactic Treatment of Migraine and Tension Headaches in Adults. Jama. 2012;307(16):1736-1745.

## Slide 13
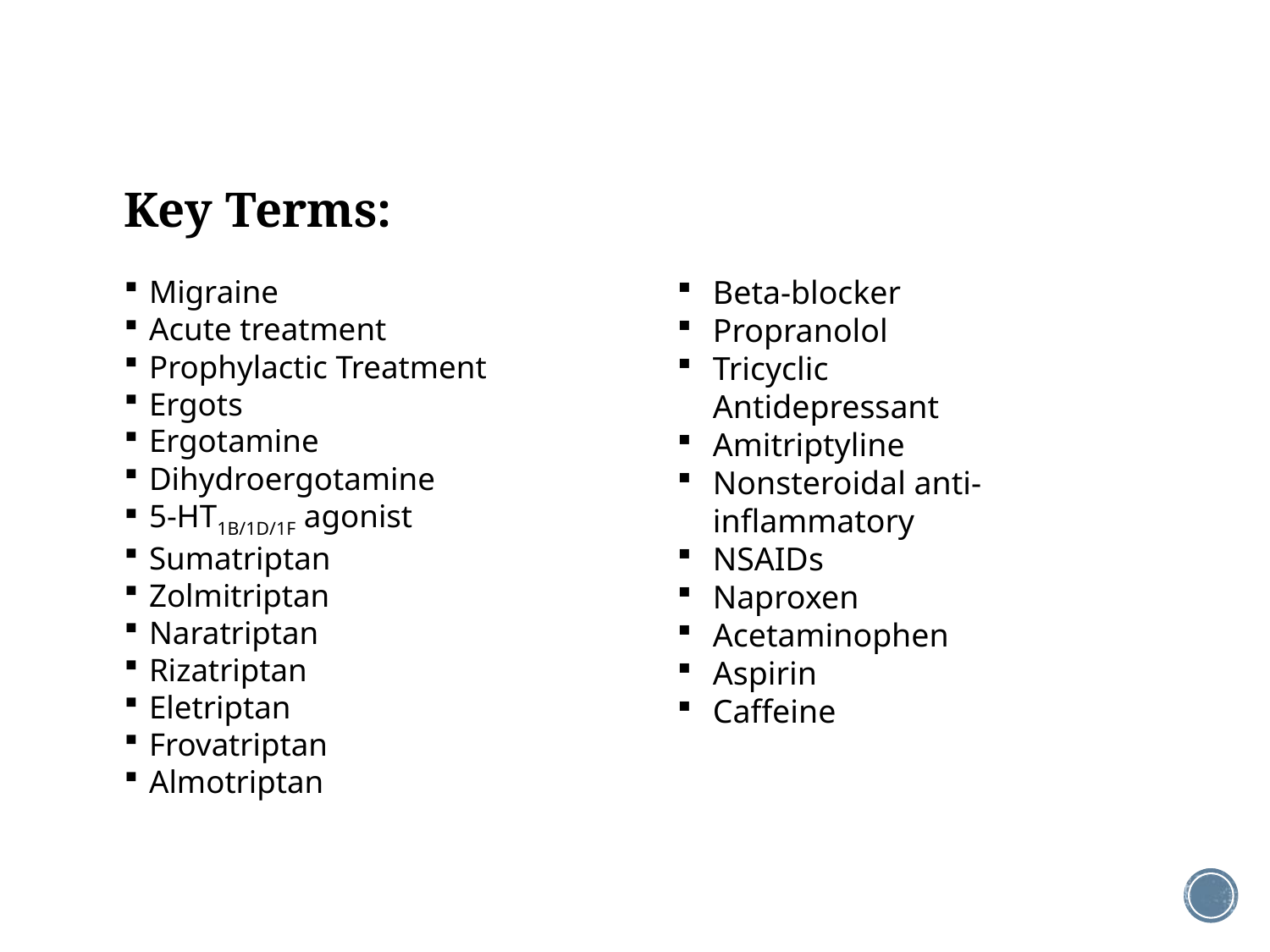

# Key Terms:
Beta-blocker
Propranolol
Tricyclic Antidepressant
Amitriptyline
Nonsteroidal anti-inflammatory
NSAIDs
Naproxen
Acetaminophen
Aspirin
Caffeine
Migraine
Acute treatment
Prophylactic Treatment
Ergots
Ergotamine
Dihydroergotamine
5-HT1B/1D/1F agonist
Sumatriptan
Zolmitriptan
Naratriptan
Rizatriptan
Eletriptan
Frovatriptan
Almotriptan
