# Supplementary material for: An Interactive Process for Delivering Pharmacologic Interventions for Migraine Headache to First-Year Medical Students
Source: MedEdPORTAL. 2020 Feb 7;16:10877. doi: 10.15766/mep_2374-8265.10877 (PMC7012313; doi:10.15766/mep_2374-8265.10877)
Supplement: Supplementary file 1 — A. Migraine Facilitator Guide.docx B. Advance Preparation Materials.docx C. Student Migraine Presentation.pptx D. Facilitator Migraine Presentation.pptx [file mep-16-10877-s001.zip › D. Facilitator Migraine Presentation.pptx]

## Slide 1
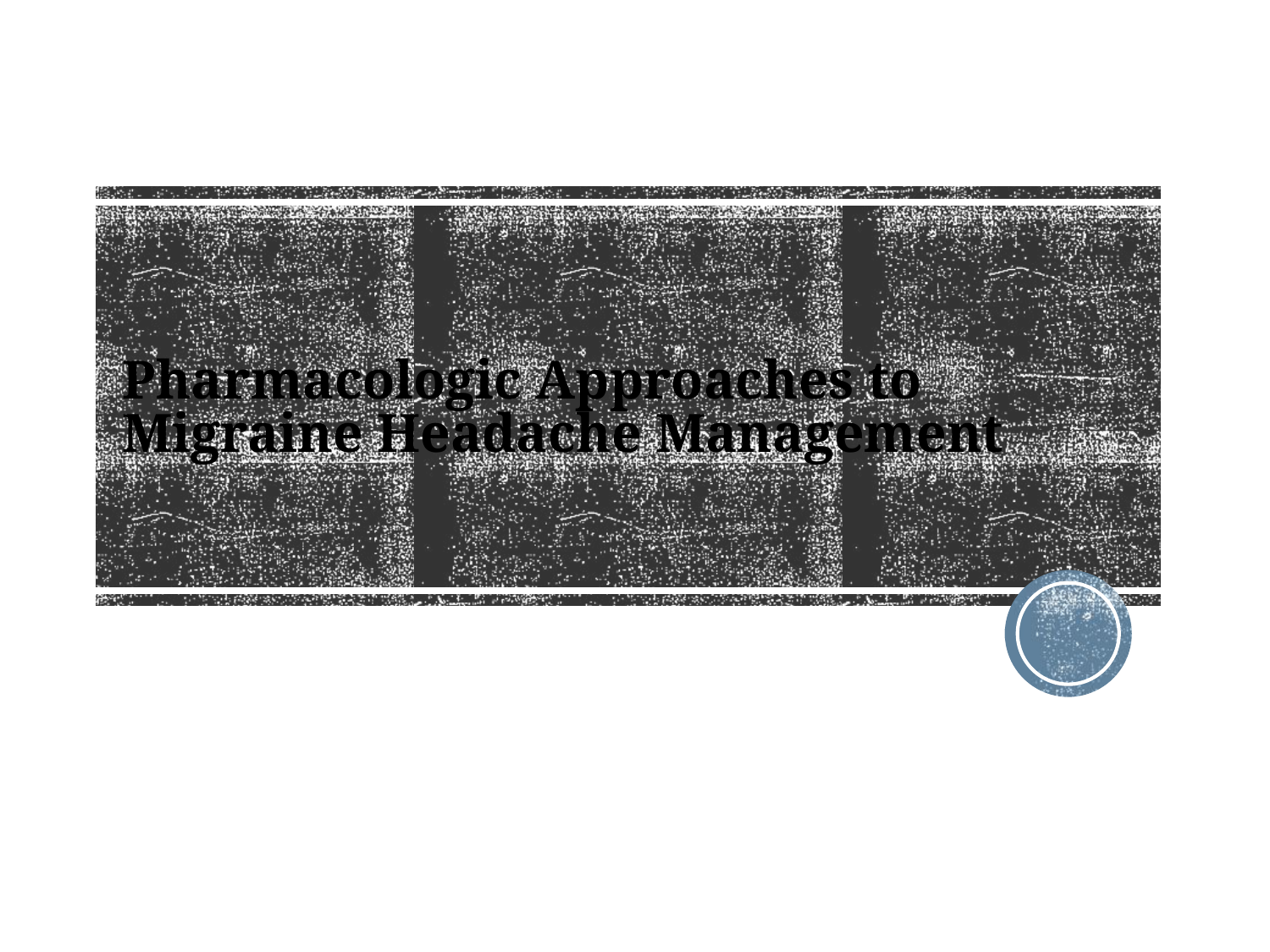

# Pharmacologic Approaches to Migraine Headache Management

## Slide 2
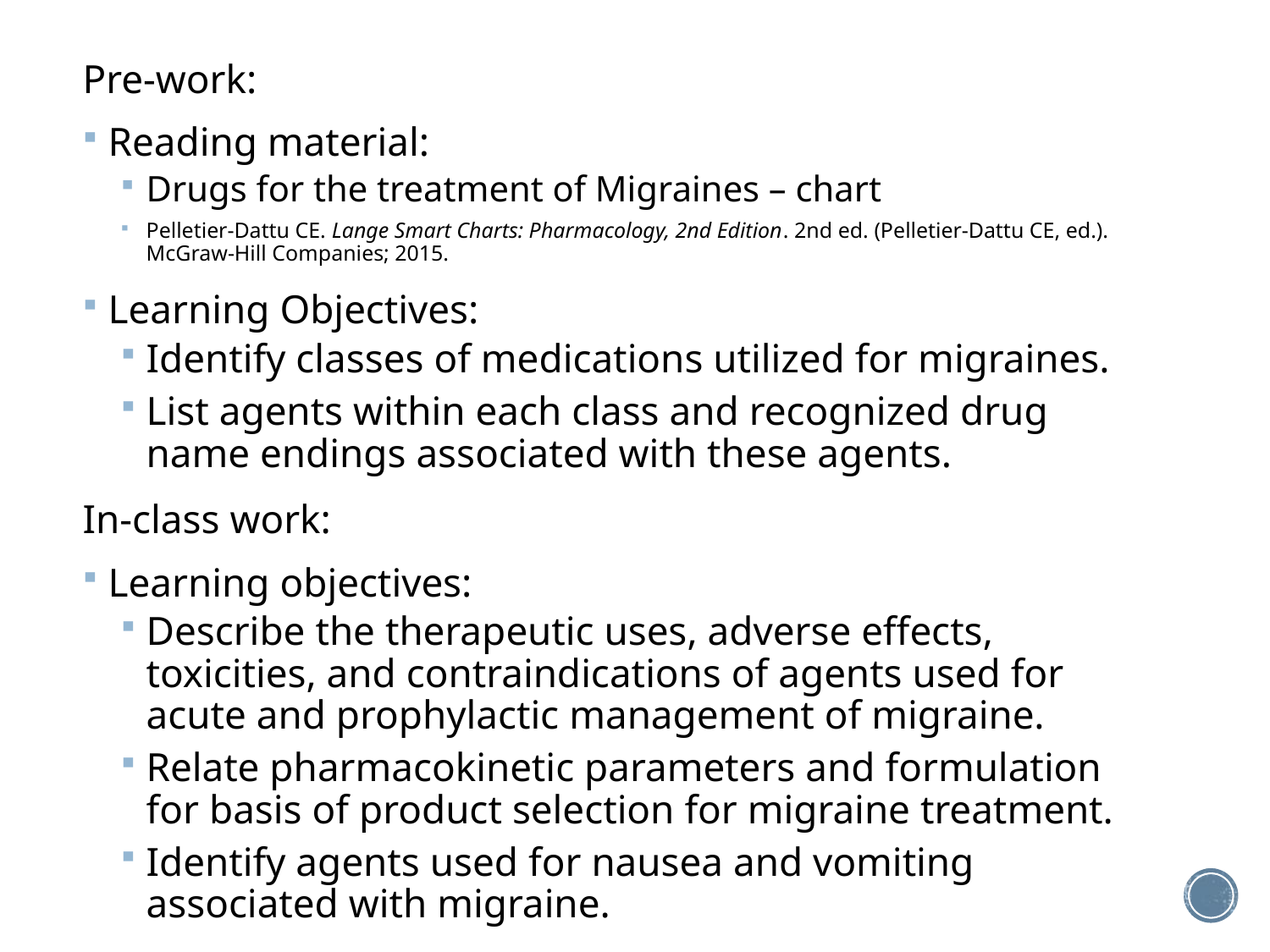

Pre-work:
Reading material:
Drugs for the treatment of Migraines – chart
Pelletier-Dattu CE. Lange Smart Charts: Pharmacology, 2nd Edition. 2nd ed. (Pelletier-Dattu CE, ed.). McGraw-Hill Companies; 2015.
Learning Objectives:
Identify classes of medications utilized for migraines.
List agents within each class and recognized drug name endings associated with these agents.
In-class work:
Learning objectives:
Describe the therapeutic uses, adverse effects, toxicities, and contraindications of agents used for acute and prophylactic management of migraine.
Relate pharmacokinetic parameters and formulation for basis of product selection for migraine treatment.
Identify agents used for nausea and vomiting associated with migraine.

## Slide 3
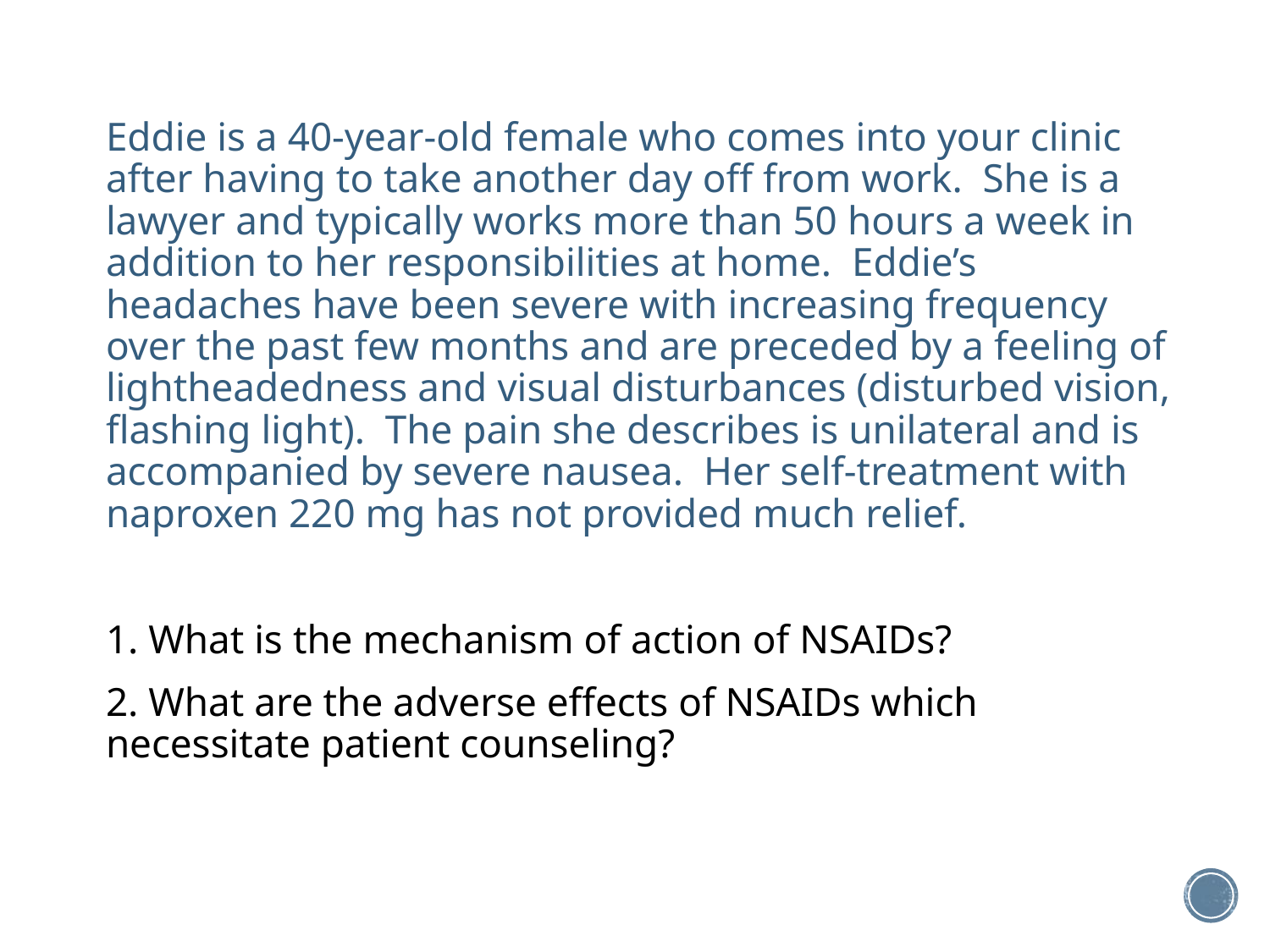

Eddie is a 40-year-old female who comes into your clinic after having to take another day off from work. She is a lawyer and typically works more than 50 hours a week in addition to her responsibilities at home. Eddie’s headaches have been severe with increasing frequency over the past few months and are preceded by a feeling of lightheadedness and visual disturbances (disturbed vision, flashing light). The pain she describes is unilateral and is accompanied by severe nausea. Her self-treatment with naproxen 220 mg has not provided much relief.
1. What is the mechanism of action of NSAIDs?
2. What are the adverse effects of NSAIDs which necessitate patient counseling?

## Slide 4
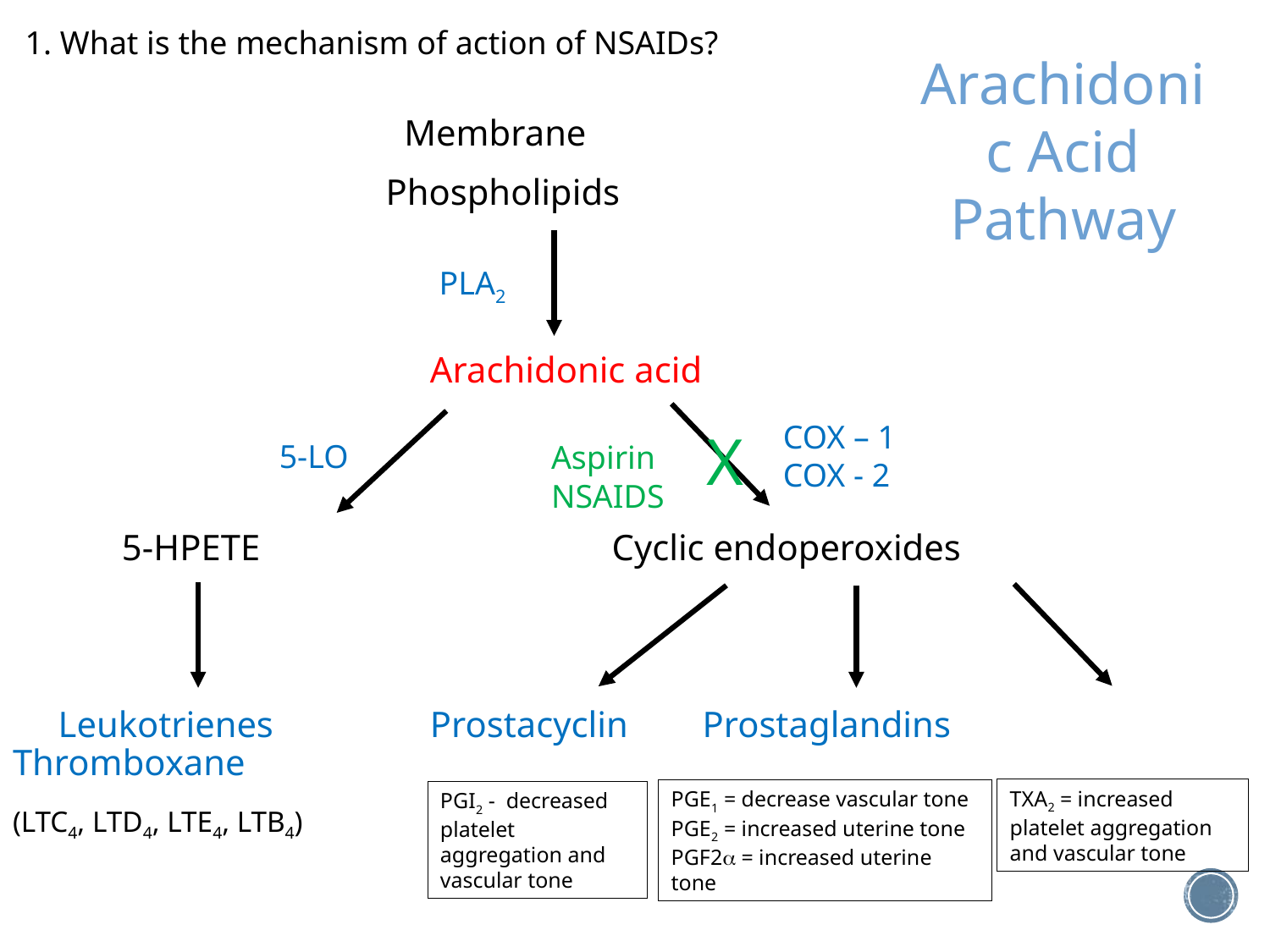

1. What is the mechanism of action of NSAIDs?
Arachidonic Acid Pathway
 Membrane
 Phospholipids
			 Arachidonic acid
 5-HPETE			 Cyclic endoperoxides
 Leukotrienes	 Prostacyclin	 Prostaglandins Thromboxane
(LTC4, LTD4, LTE4, LTB4)
PLA2
COX – 1
COX - 2
X
5-LO
Aspirin
NSAIDS
TXA2 = increased platelet aggregation and vascular tone
PGE1 = decrease vascular tone
PGE2 = increased uterine tone
PGF2α = increased uterine tone
PGI2 - decreased platelet aggregation and vascular tone

## Slide 5
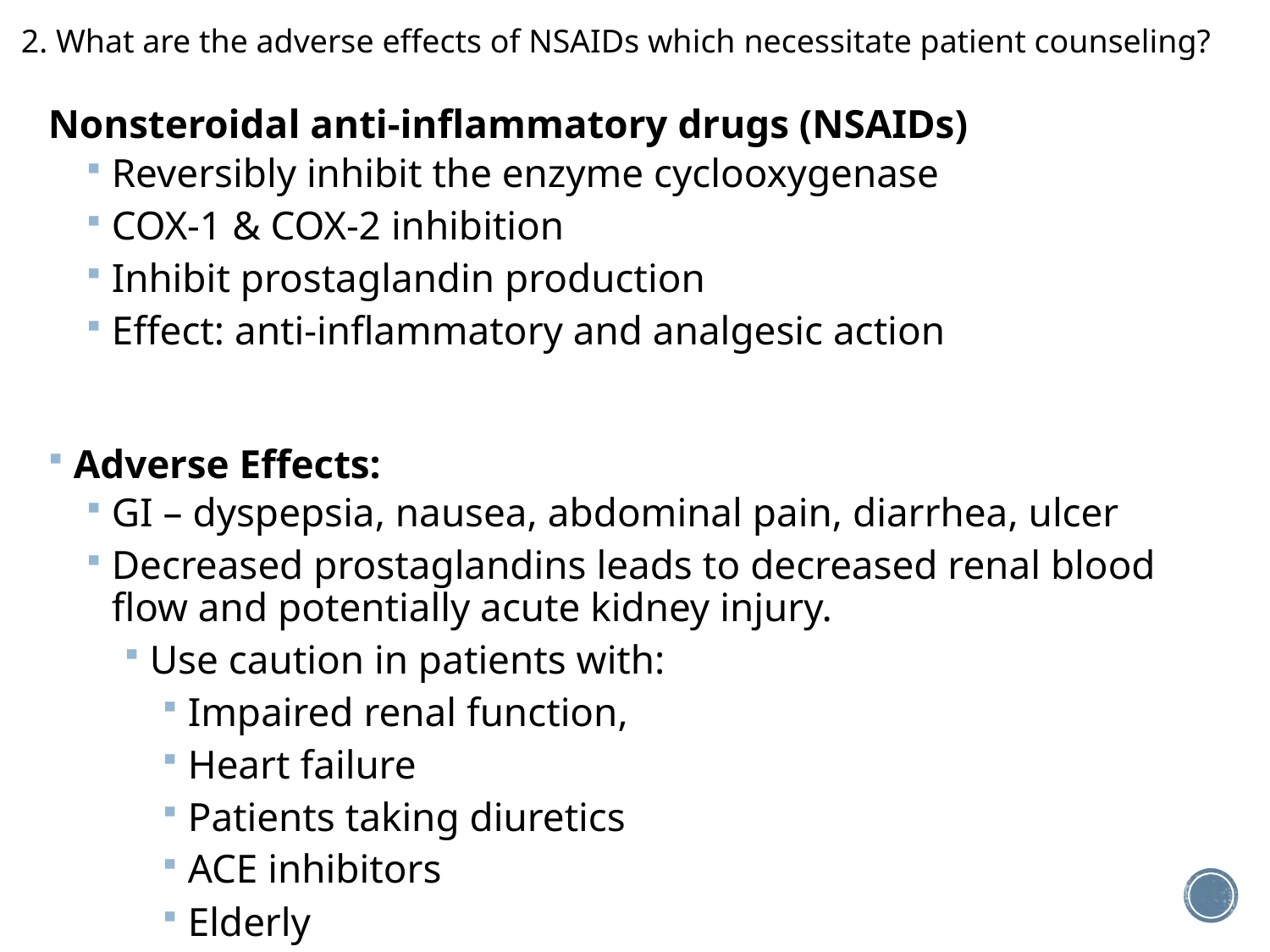

2. What are the adverse effects of NSAIDs which necessitate patient counseling?
Nonsteroidal anti-inflammatory drugs (NSAIDs)
Reversibly inhibit the enzyme cyclooxygenase
COX-1 & COX-2 inhibition
Inhibit prostaglandin production
Effect: anti-inflammatory and analgesic action
Adverse Effects:
GI – dyspepsia, nausea, abdominal pain, diarrhea, ulcer
Decreased prostaglandins leads to decreased renal blood flow and potentially acute kidney injury.
Use caution in patients with:
Impaired renal function,
Heart failure
Patients taking diuretics
ACE inhibitors
Elderly

## Slide 6
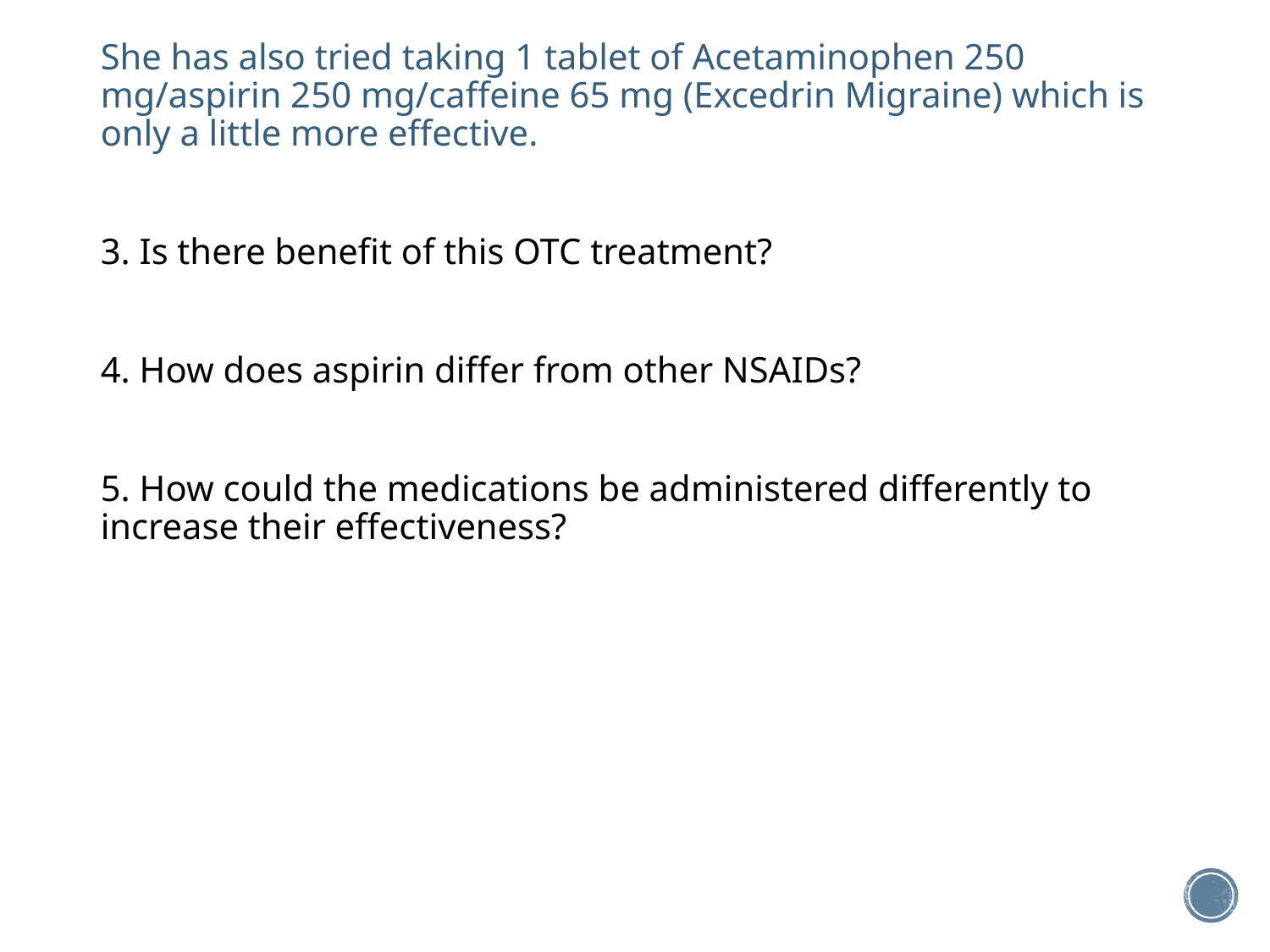

She has also tried taking 1 tablet of Acetaminophen 250 mg/aspirin 250 mg/caffeine 65 mg (Excedrin Migraine) which is only a little more effective.
3. Is there benefit of this OTC treatment?
4. How does aspirin differ from other NSAIDs?
5. How could the medications be administered differently to increase their effectiveness?

## Slide 7
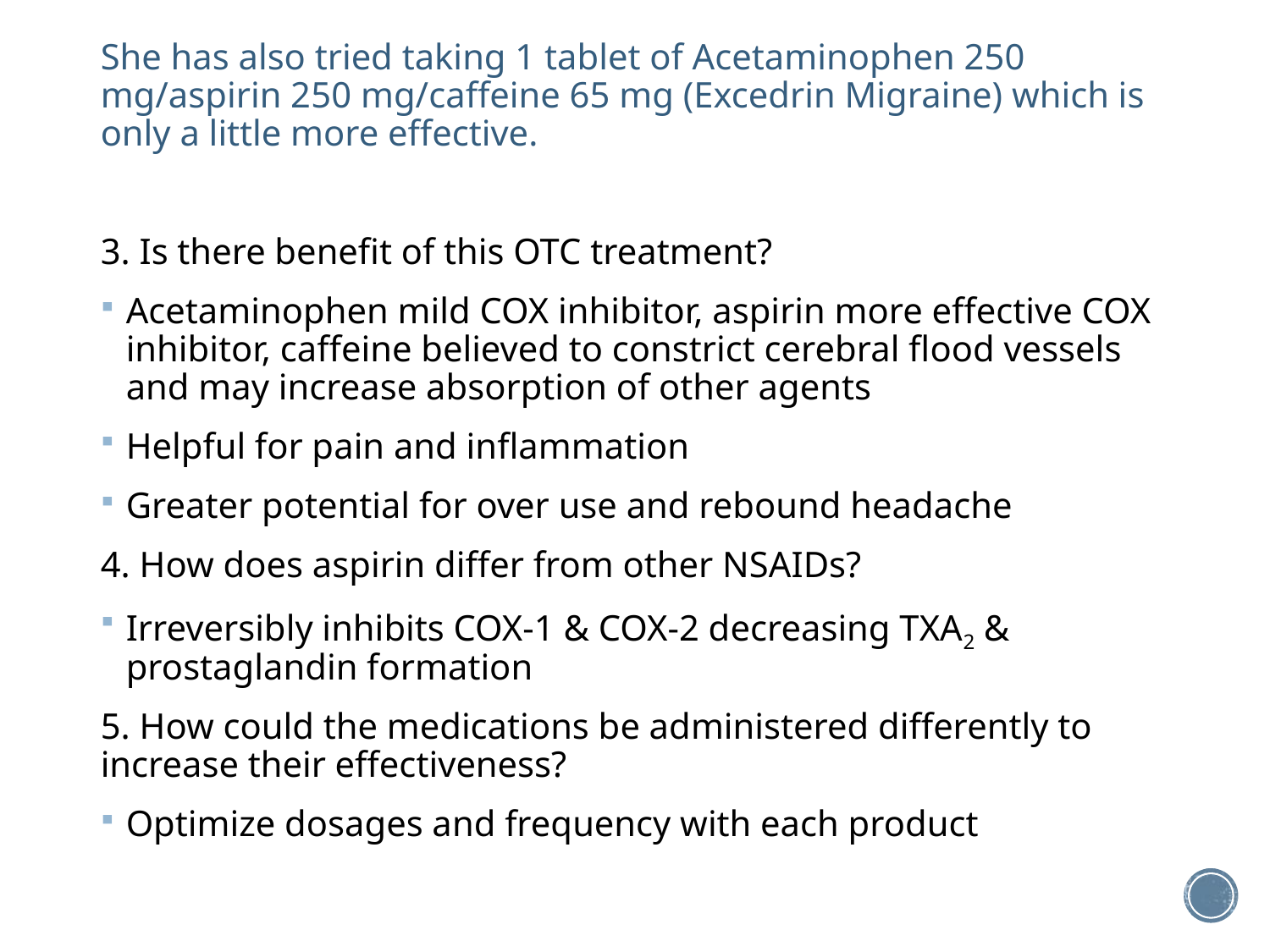

She has also tried taking 1 tablet of Acetaminophen 250 mg/aspirin 250 mg/caffeine 65 mg (Excedrin Migraine) which is only a little more effective.
3. Is there benefit of this OTC treatment?
Acetaminophen mild COX inhibitor, aspirin more effective COX inhibitor, caffeine believed to constrict cerebral flood vessels and may increase absorption of other agents
Helpful for pain and inflammation
Greater potential for over use and rebound headache
4. How does aspirin differ from other NSAIDs?
Irreversibly inhibits COX-1 & COX-2 decreasing TXA2 & prostaglandin formation
5. How could the medications be administered differently to increase their effectiveness?
Optimize dosages and frequency with each product

## Slide 8
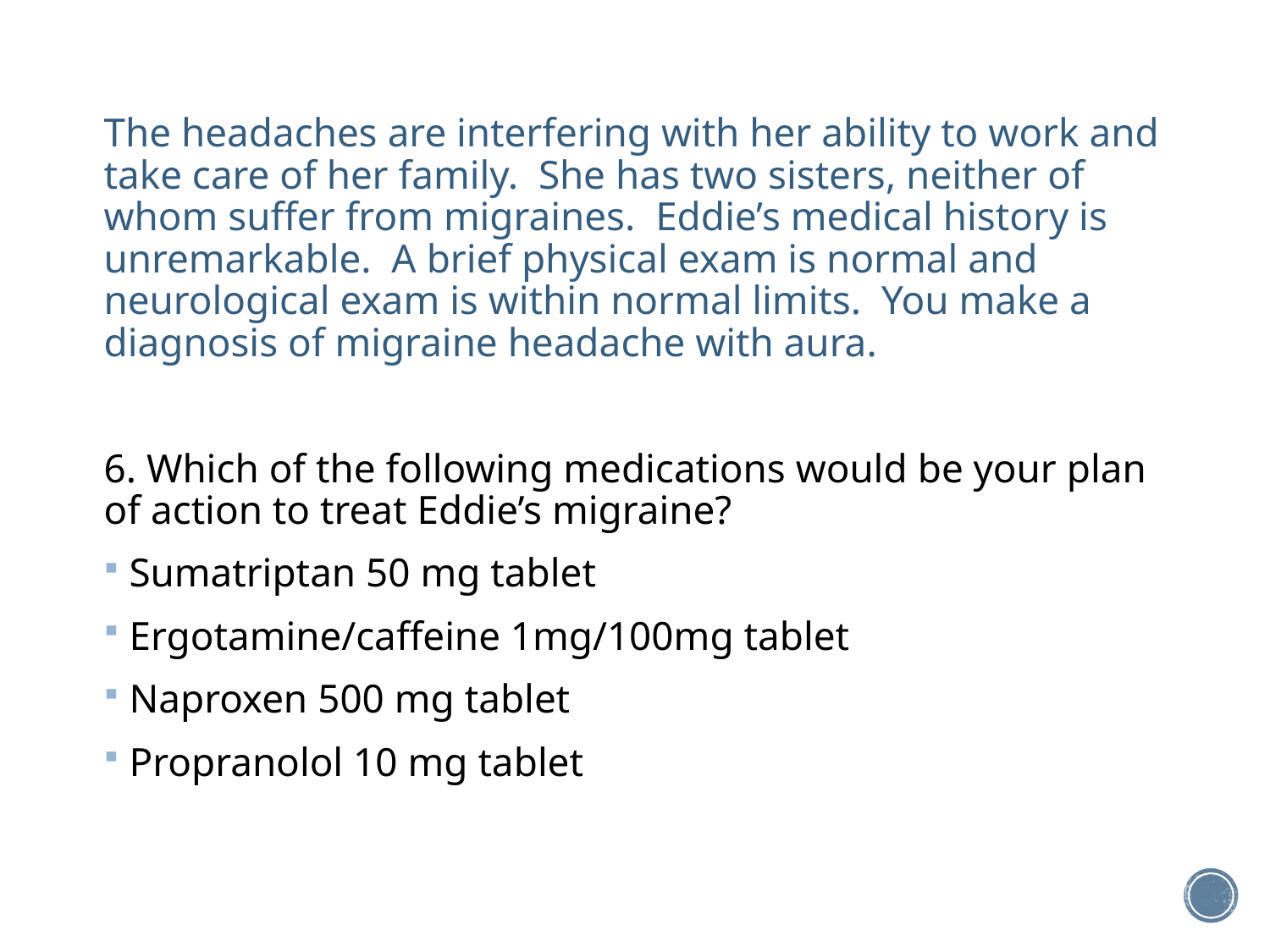

The headaches are interfering with her ability to work and take care of her family. She has two sisters, neither of whom suffer from migraines. Eddie’s medical history is unremarkable. A brief physical exam is normal and neurological exam is within normal limits. You make a diagnosis of migraine headache with aura.
6. Which of the following medications would be your plan of action to treat Eddie’s migraine?
Sumatriptan 50 mg tablet
Ergotamine/caffeine 1mg/100mg tablet
Naproxen 500 mg tablet
Propranolol 10 mg tablet

## Slide 9
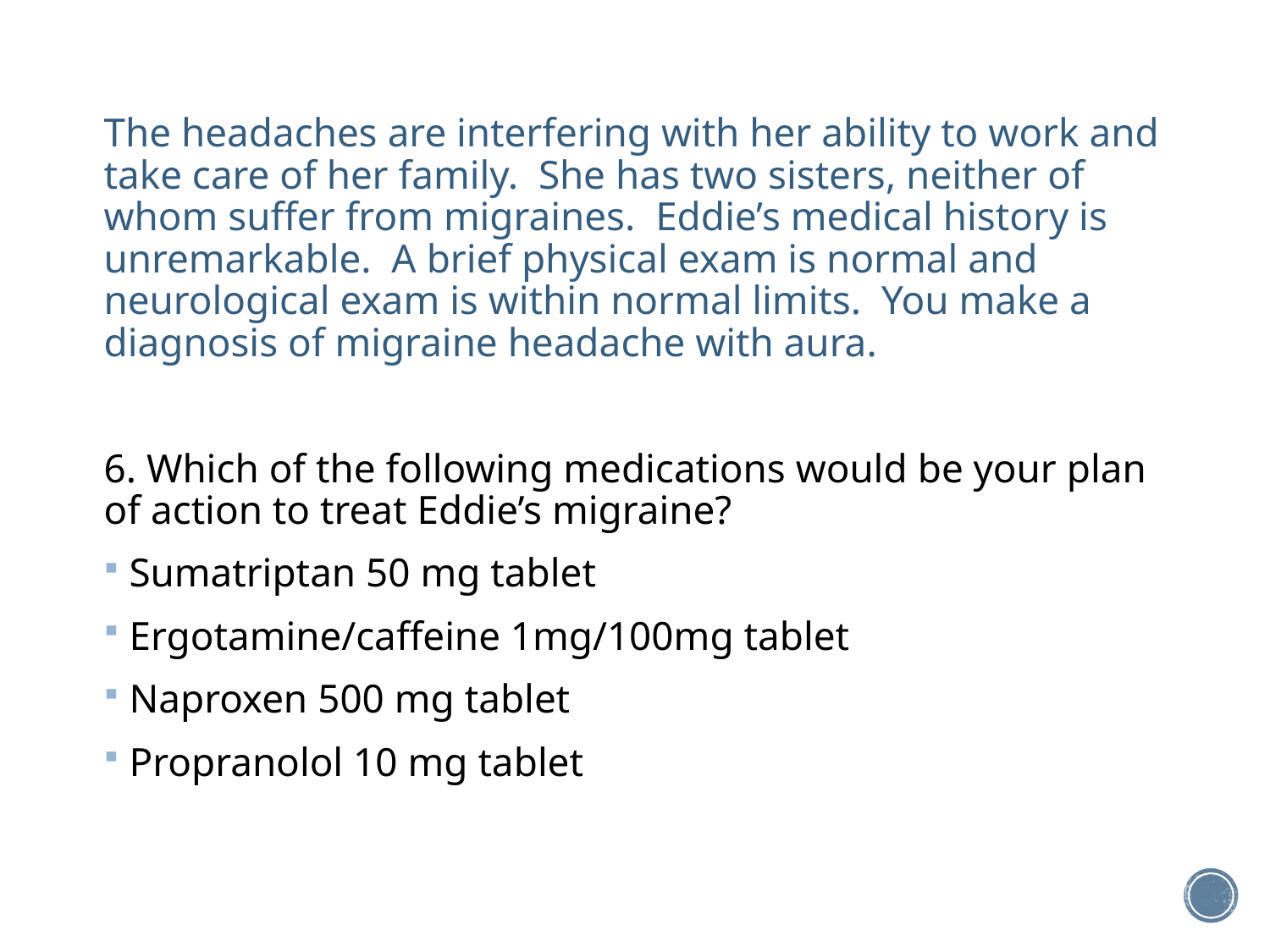

The headaches are interfering with her ability to work and take care of her family. She has two sisters, neither of whom suffer from migraines. Eddie’s medical history is unremarkable. A brief physical exam is normal and neurological exam is within normal limits. You make a diagnosis of migraine headache with aura.
6. Which of the following medications would be your plan of action to treat Eddie’s migraine?
Sumatriptan 50 mg tablet
Ergotamine/caffeine 1mg/100mg tablet
Naproxen 500 mg tablet
Propranolol 10 mg tablet

## Slide 10
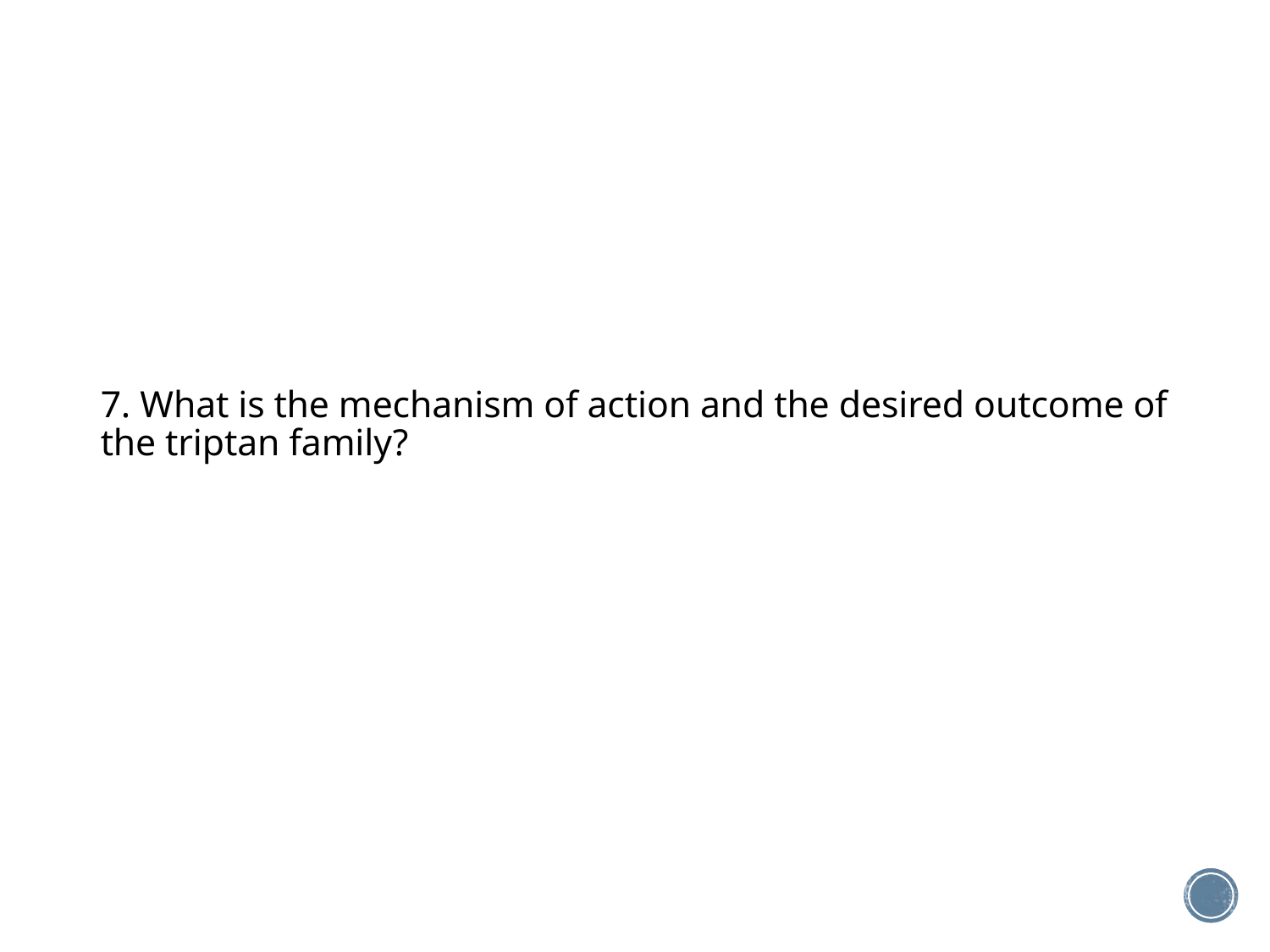

7. What is the mechanism of action and the desired outcome of the triptan family?

## Slide 11
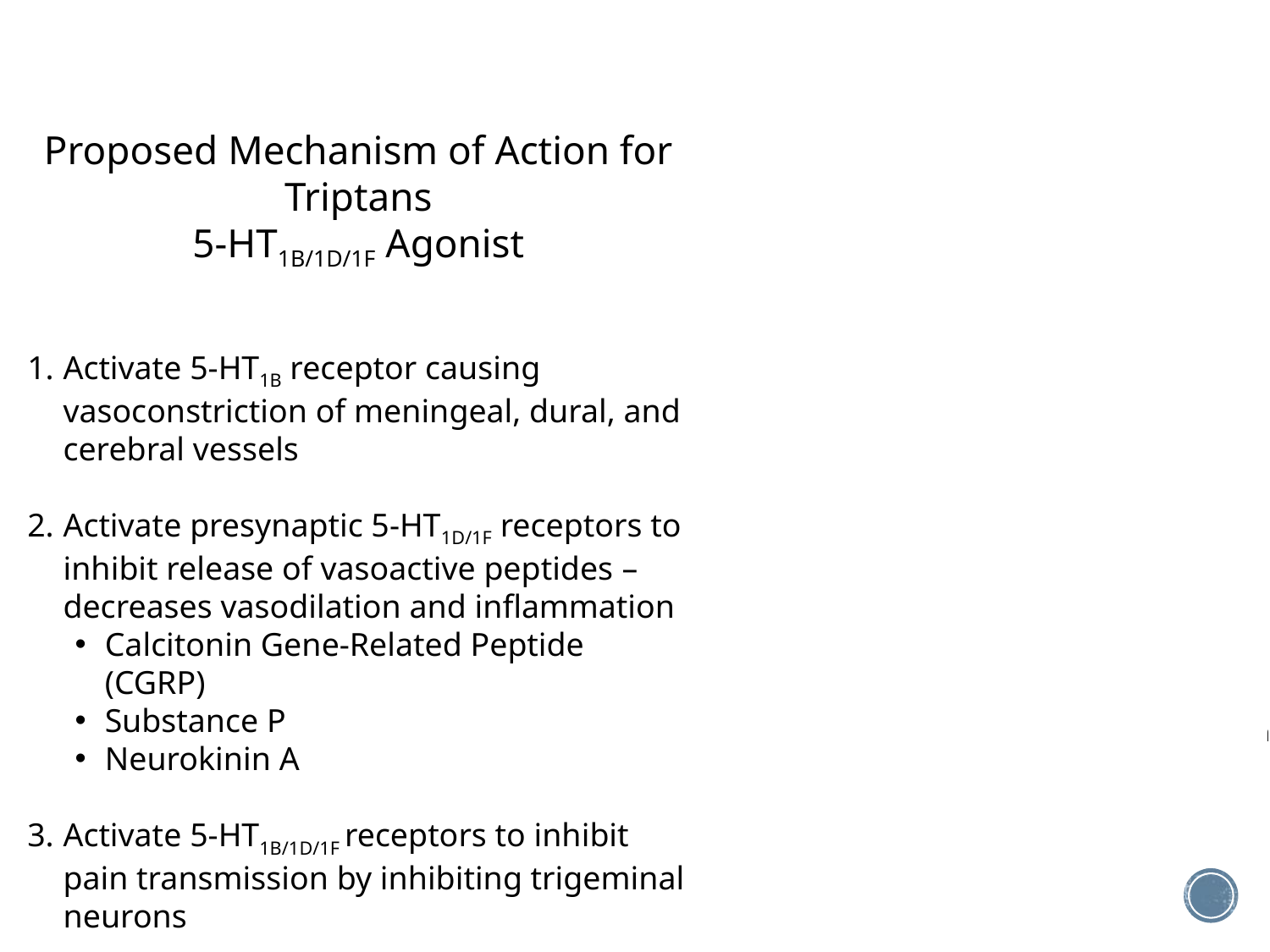

7. What is the mechanism of action and the desired outcome of the triptan family?
Proposed Mechanism of Action for Triptans
5-HT1B/1D/1F Agonist
Activate 5-HT1B receptor causing vasoconstriction of meningeal, dural, and cerebral vessels
Activate presynaptic 5-HT1D/1F receptors to inhibit release of vasoactive peptides – decreases vasodilation and inflammation
Calcitonin Gene-Related Peptide (CGRP)
Substance P
Neurokinin A
Activate 5-HT1B/1D/1F receptors to inhibit pain transmission by inhibiting trigeminal neurons
Trigeminal Nerve
TRIPTANS
5-HT1D
Inhibition of NT release
5-HT1F
Nociceptive & Inflammatory Neurotransmitters
CGRP
NKA
Substance P
5-HT1B
Vasoconstriction
-

## Slide 12
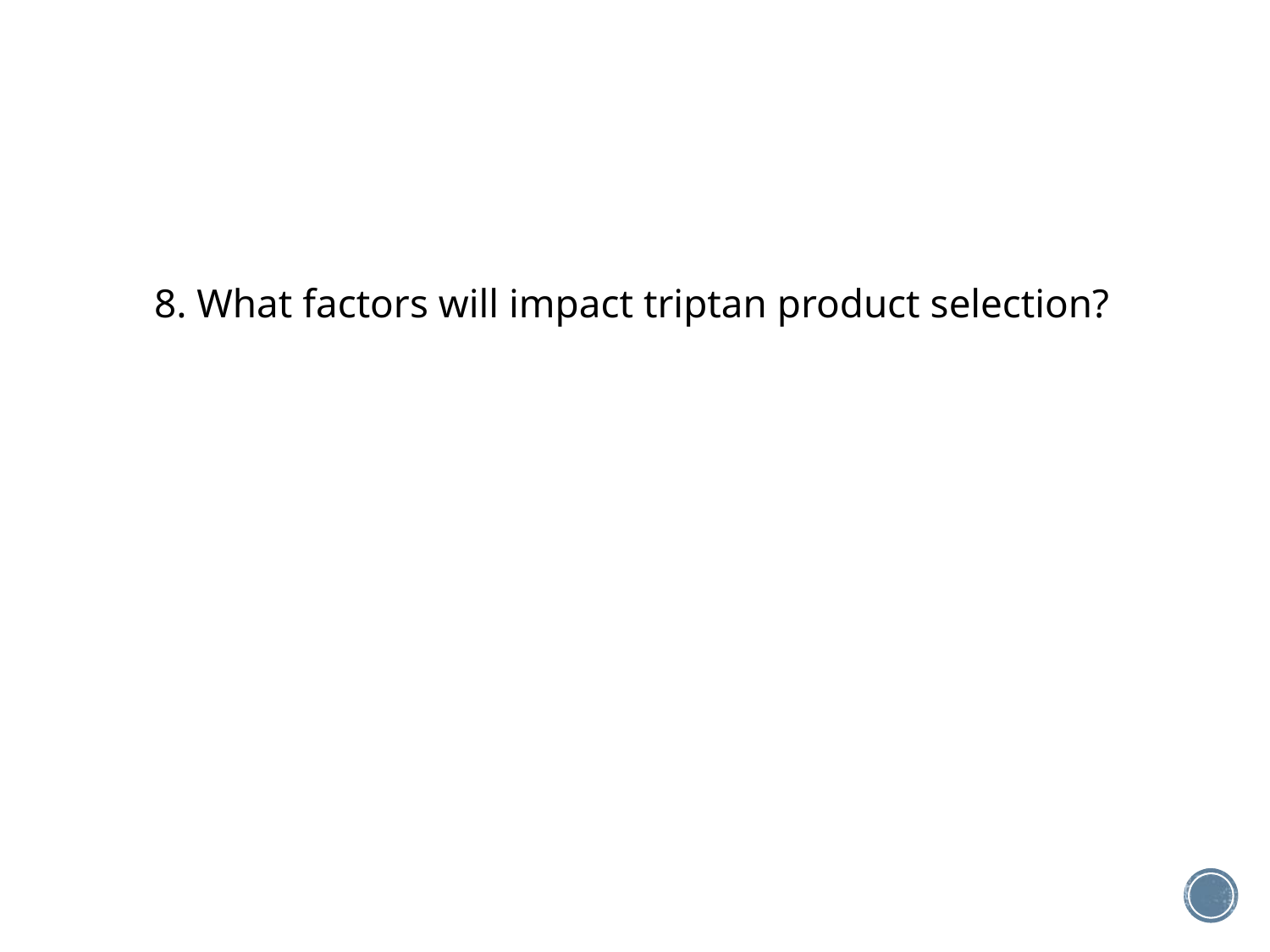

8. What factors will impact triptan product selection?

## Slide 13
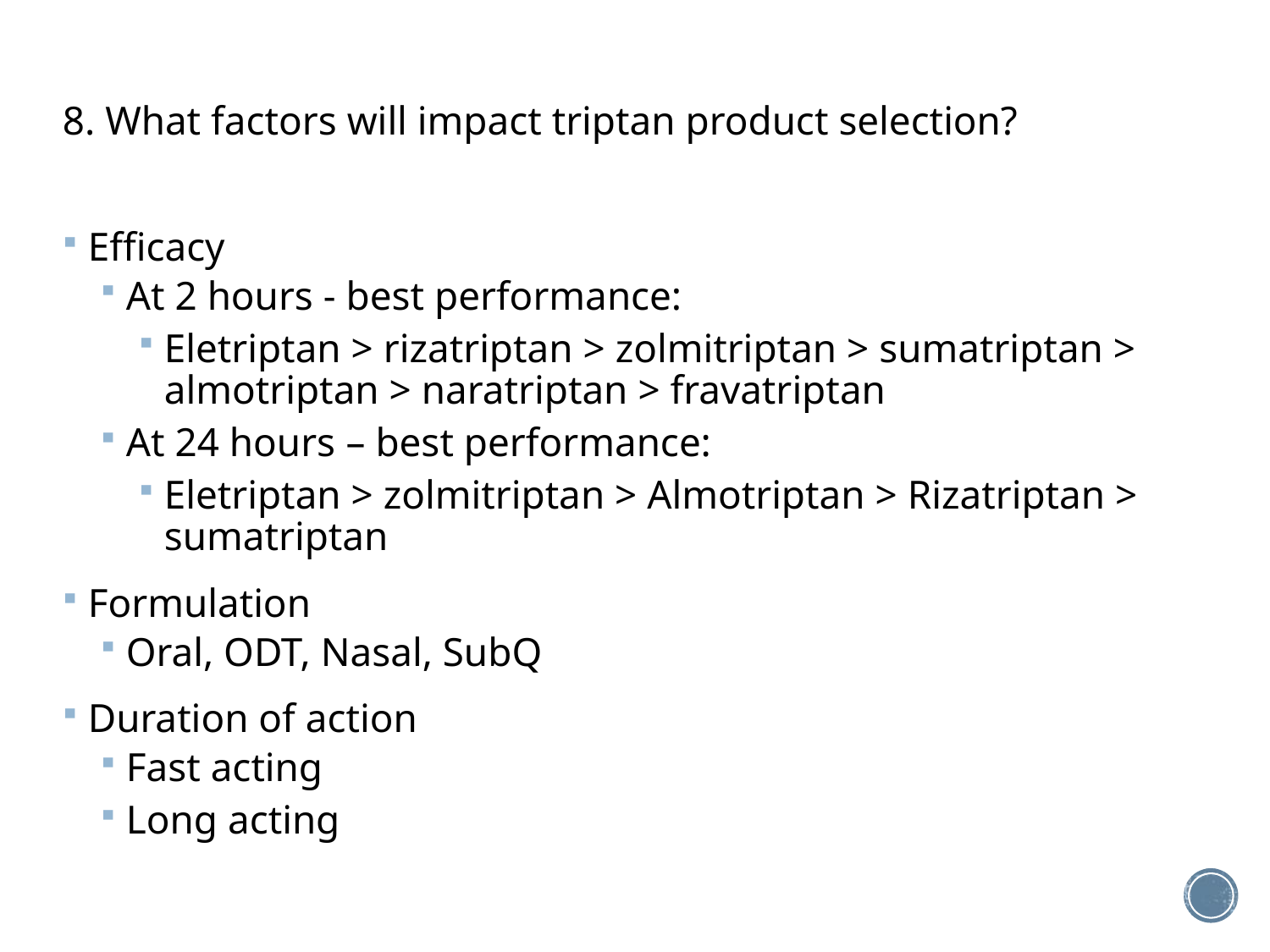

8. What factors will impact triptan product selection?
Efficacy
At 2 hours - best performance:
Eletriptan > rizatriptan > zolmitriptan > sumatriptan > almotriptan > naratriptan > fravatriptan
At 24 hours – best performance:
Eletriptan > zolmitriptan > Almotriptan > Rizatriptan > sumatriptan
Formulation
Oral, ODT, Nasal, SubQ
Duration of action
Fast acting
Long acting

## Slide 14
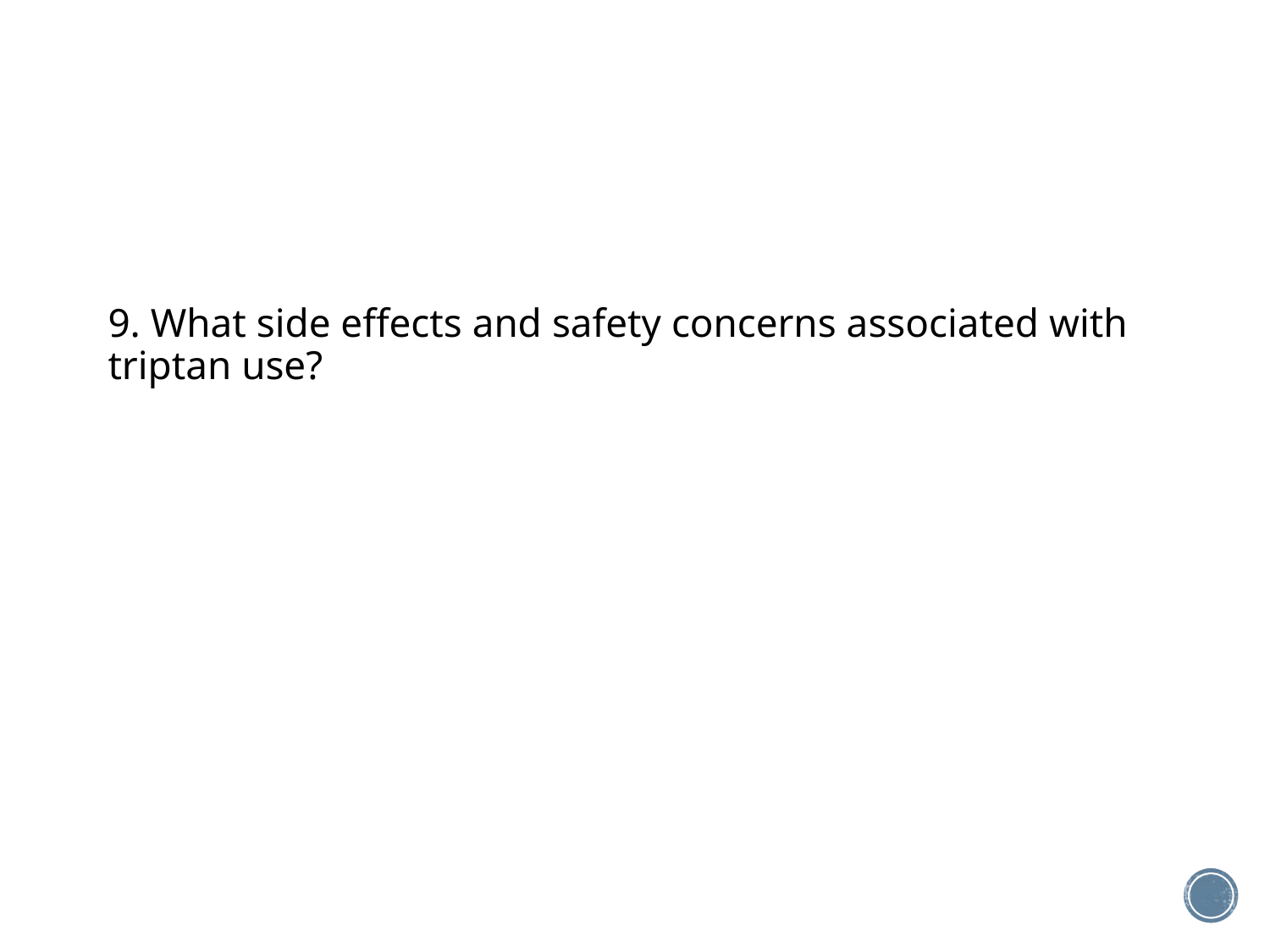

9. What side effects and safety concerns associated with triptan use?

## Slide 15
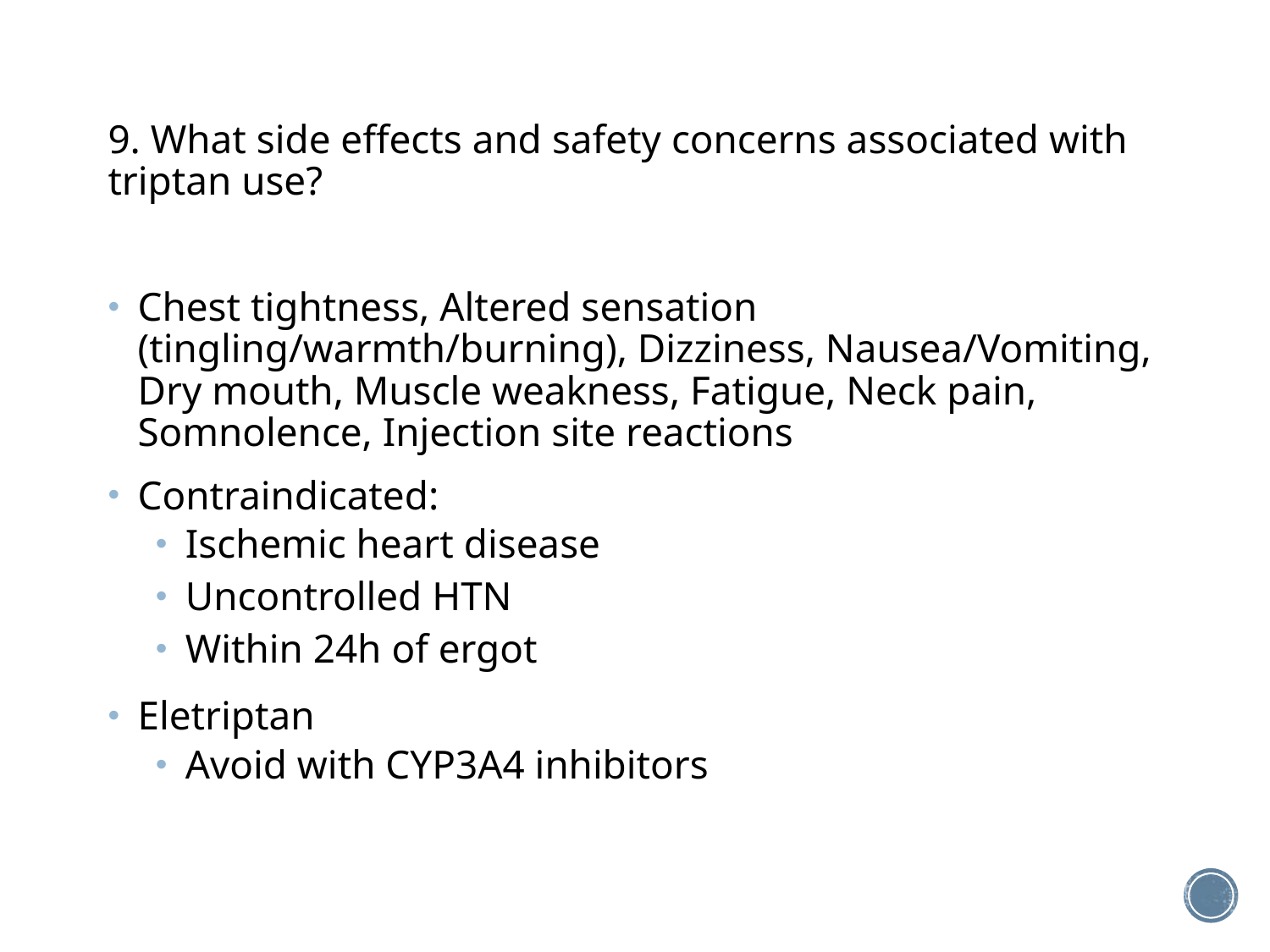

9. What side effects and safety concerns associated with triptan use?
Chest tightness, Altered sensation (tingling/warmth/burning), Dizziness, Nausea/Vomiting, Dry mouth, Muscle weakness, Fatigue, Neck pain, Somnolence, Injection site reactions
Contraindicated:
Ischemic heart disease
Uncontrolled HTN
Within 24h of ergot
Eletriptan
Avoid with CYP3A4 inhibitors

## Slide 16
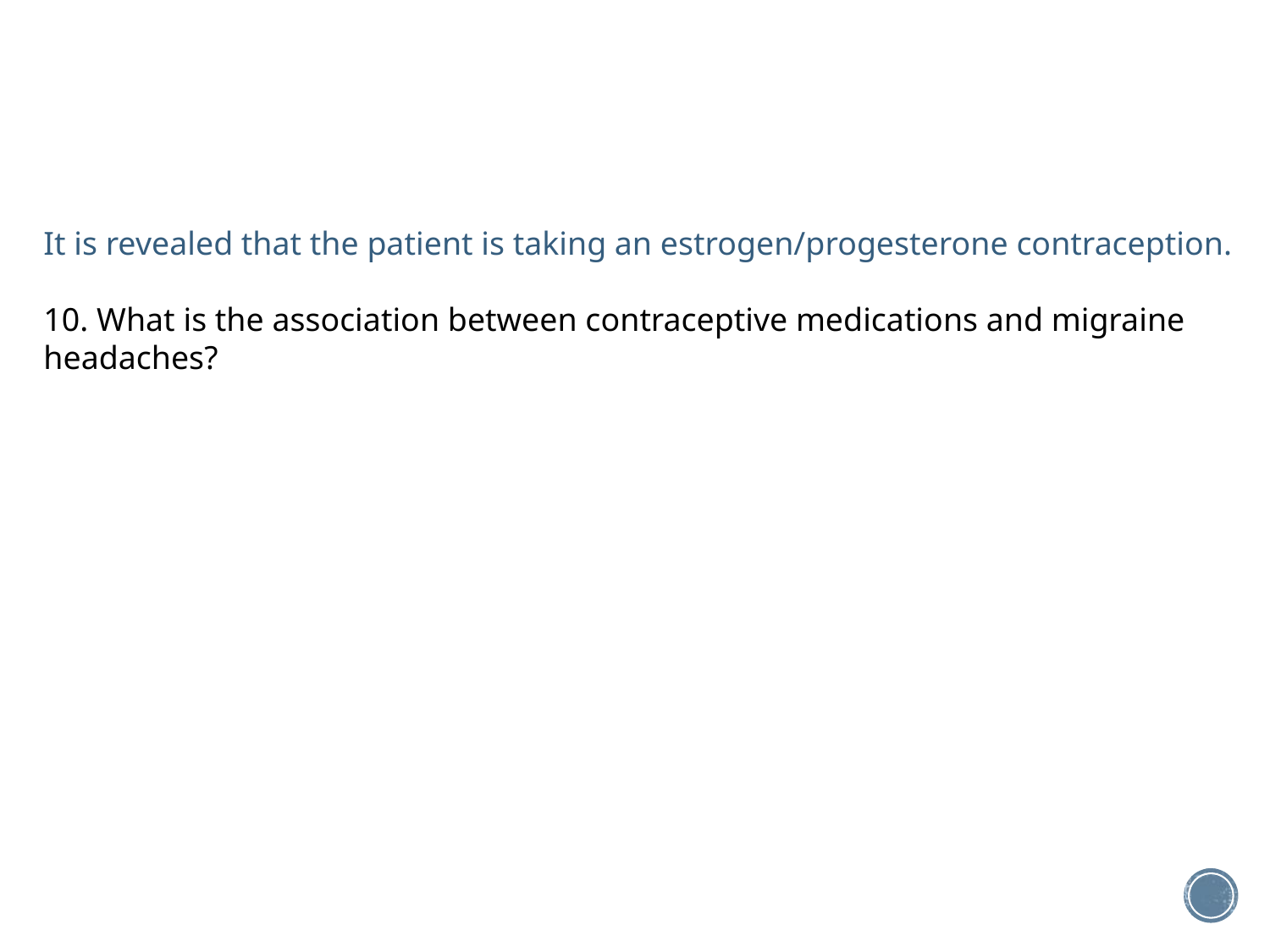

It is revealed that the patient is taking an estrogen/progesterone contraception.
10. What is the association between contraceptive medications and migraine headaches?

## Slide 17
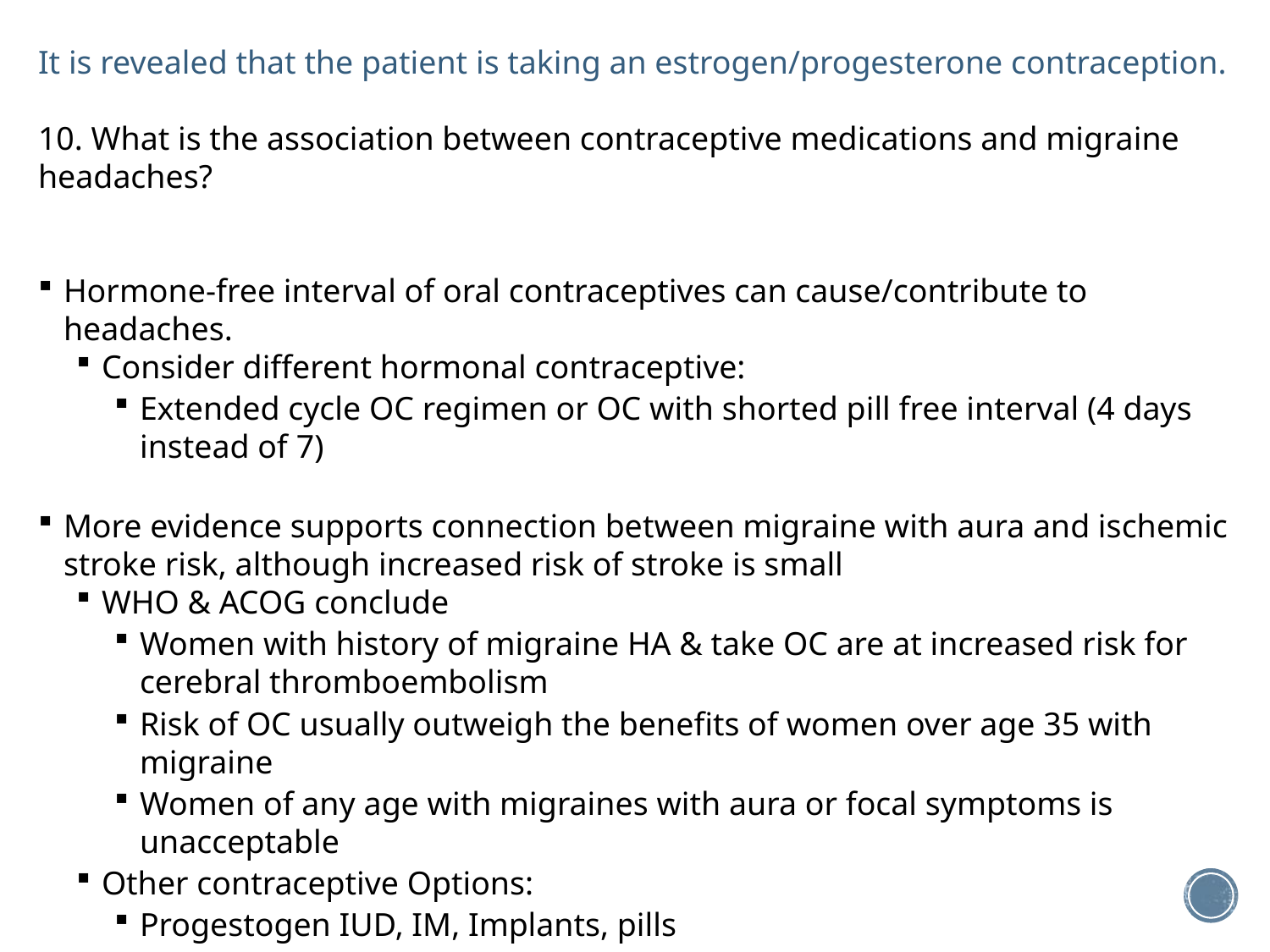

It is revealed that the patient is taking an estrogen/progesterone contraception.
10. What is the association between contraceptive medications and migraine headaches?
Hormone-free interval of oral contraceptives can cause/contribute to headaches.
Consider different hormonal contraceptive:
Extended cycle OC regimen or OC with shorted pill free interval (4 days instead of 7)
More evidence supports connection between migraine with aura and ischemic stroke risk, although increased risk of stroke is small
WHO & ACOG conclude
Women with history of migraine HA & take OC are at increased risk for cerebral thromboembolism
Risk of OC usually outweigh the benefits of women over age 35 with migraine
Women of any age with migraines with aura or focal symptoms is unacceptable
Other contraceptive Options:
Progestogen IUD, IM, Implants, pills

## Slide 18
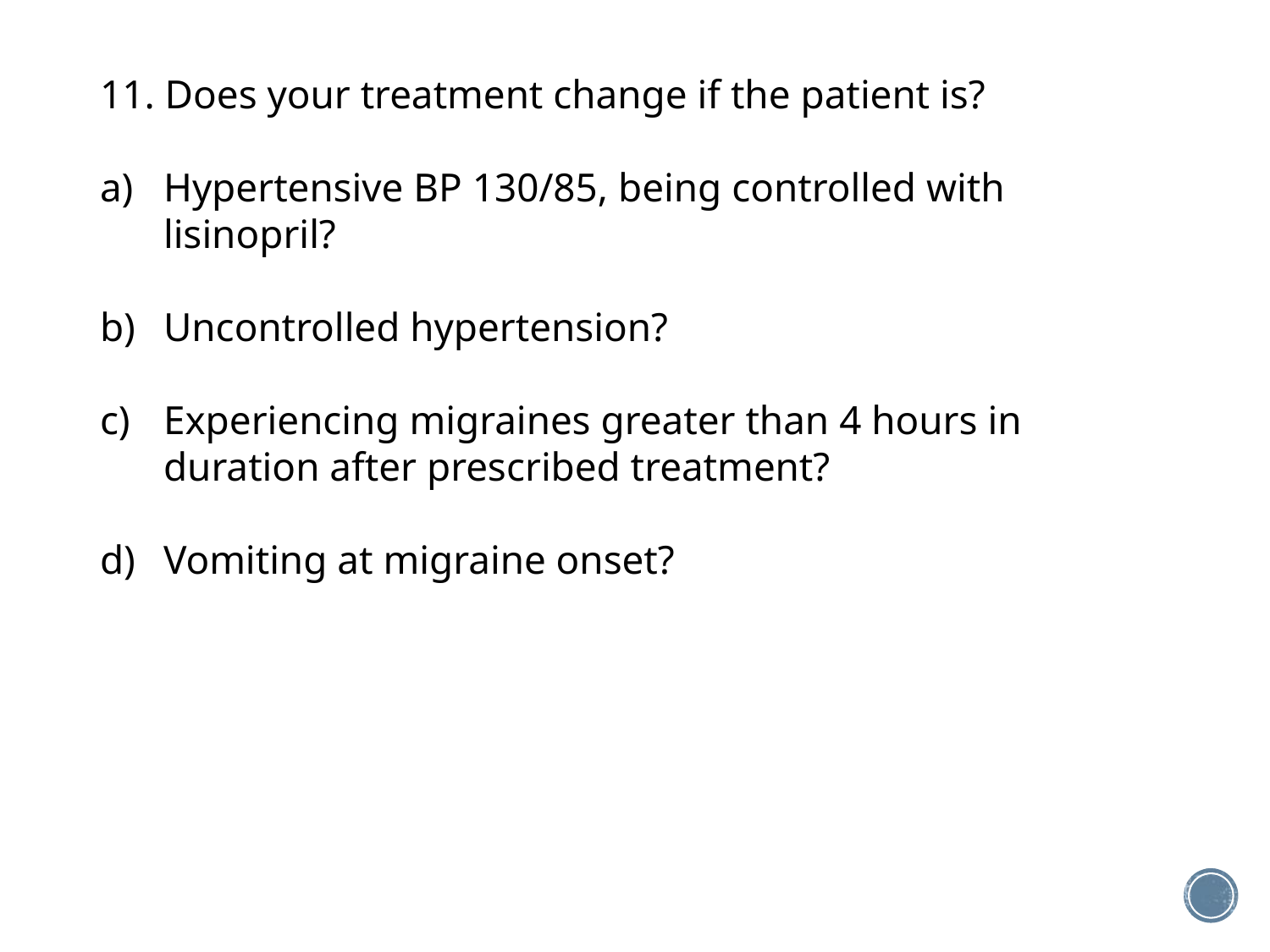

11. Does your treatment change if the patient is?
Hypertensive BP 130/85, being controlled with lisinopril?
Uncontrolled hypertension?
Experiencing migraines greater than 4 hours in duration after prescribed treatment?
Vomiting at migraine onset?

## Slide 19
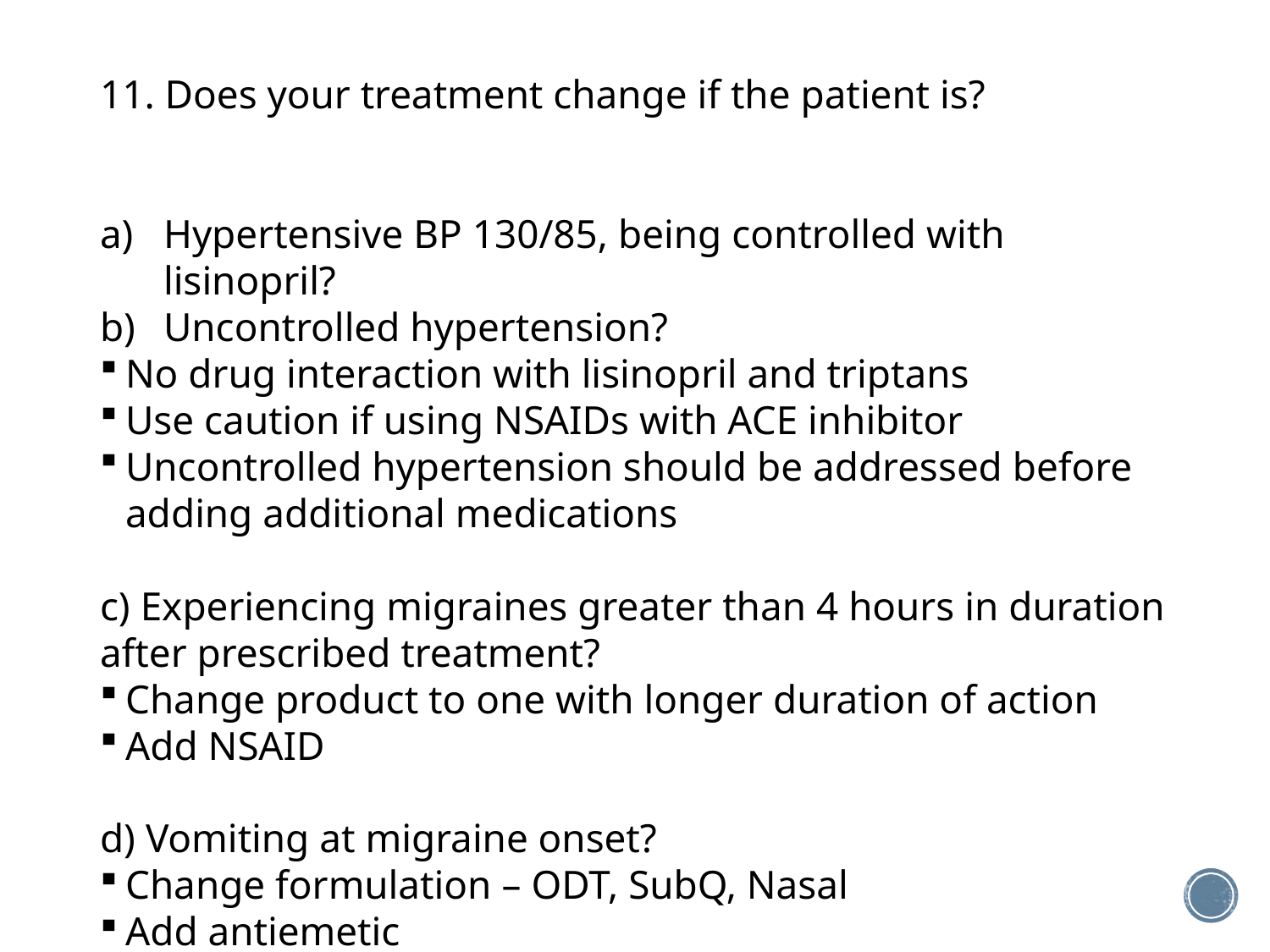

11. Does your treatment change if the patient is?
Hypertensive BP 130/85, being controlled with lisinopril?
Uncontrolled hypertension?
No drug interaction with lisinopril and triptans
Use caution if using NSAIDs with ACE inhibitor
Uncontrolled hypertension should be addressed before adding additional medications
c) Experiencing migraines greater than 4 hours in duration after prescribed treatment?
Change product to one with longer duration of action
Add NSAID
d) Vomiting at migraine onset?
Change formulation – ODT, SubQ, Nasal
Add antiemetic

## Slide 20
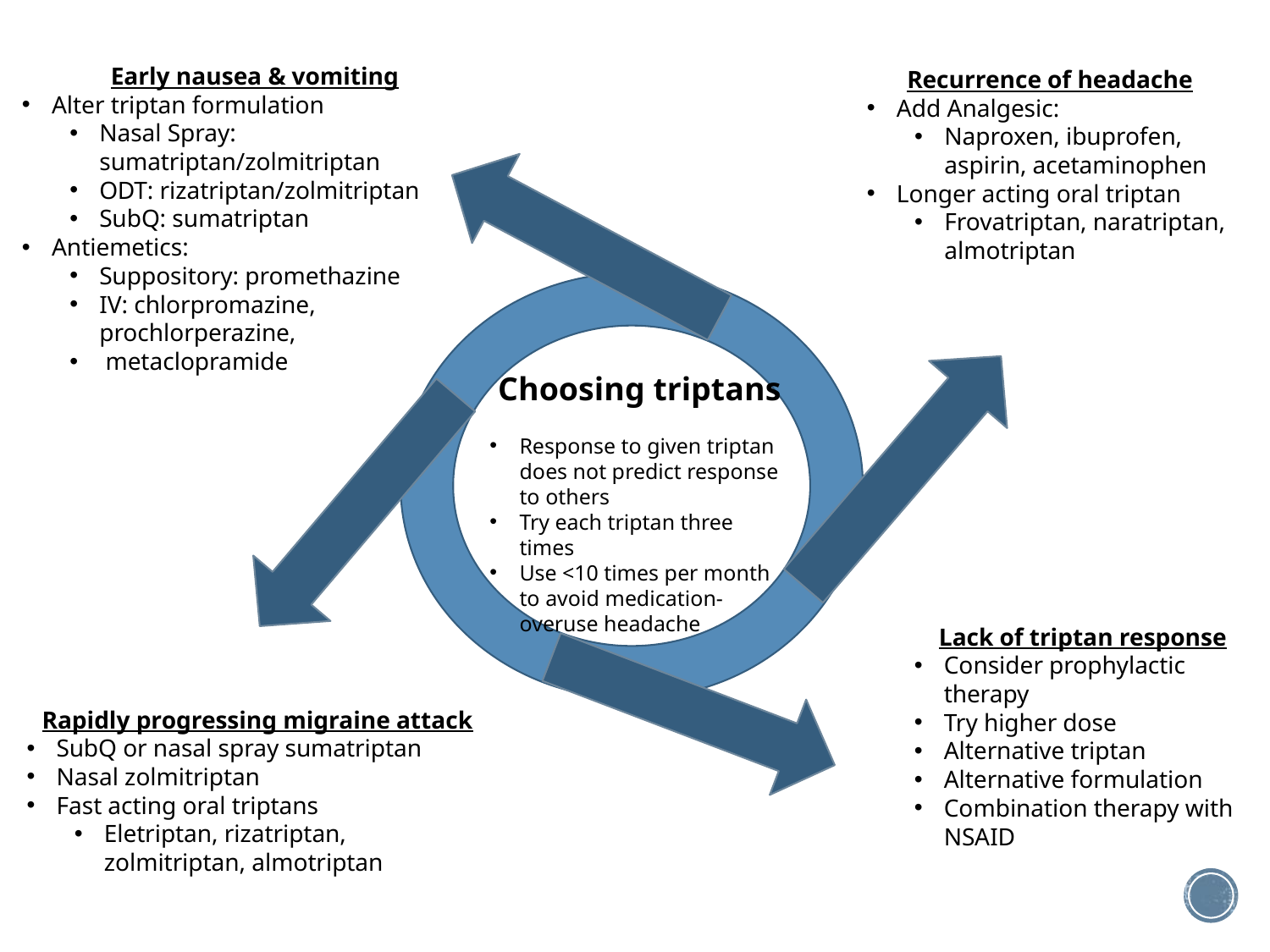

Early nausea & vomiting
Alter triptan formulation
Nasal Spray: sumatriptan/zolmitriptan
ODT: rizatriptan/zolmitriptan
SubQ: sumatriptan
Antiemetics:
Suppository: promethazine
IV: chlorpromazine, prochlorperazine,
 metaclopramide
Recurrence of headache
Add Analgesic:
Naproxen, ibuprofen, aspirin, acetaminophen
Longer acting oral triptan
Frovatriptan, naratriptan, almotriptan
Choosing triptans
Response to given triptan does not predict response to others
Try each triptan three times
Use <10 times per month to avoid medication-overuse headache
Lack of triptan response
Consider prophylactic therapy
Try higher dose
Alternative triptan
Alternative formulation
Combination therapy with NSAID
Rapidly progressing migraine attack
SubQ or nasal spray sumatriptan
Nasal zolmitriptan
Fast acting oral triptans
Eletriptan, rizatriptan, zolmitriptan, almotriptan

## Slide 21
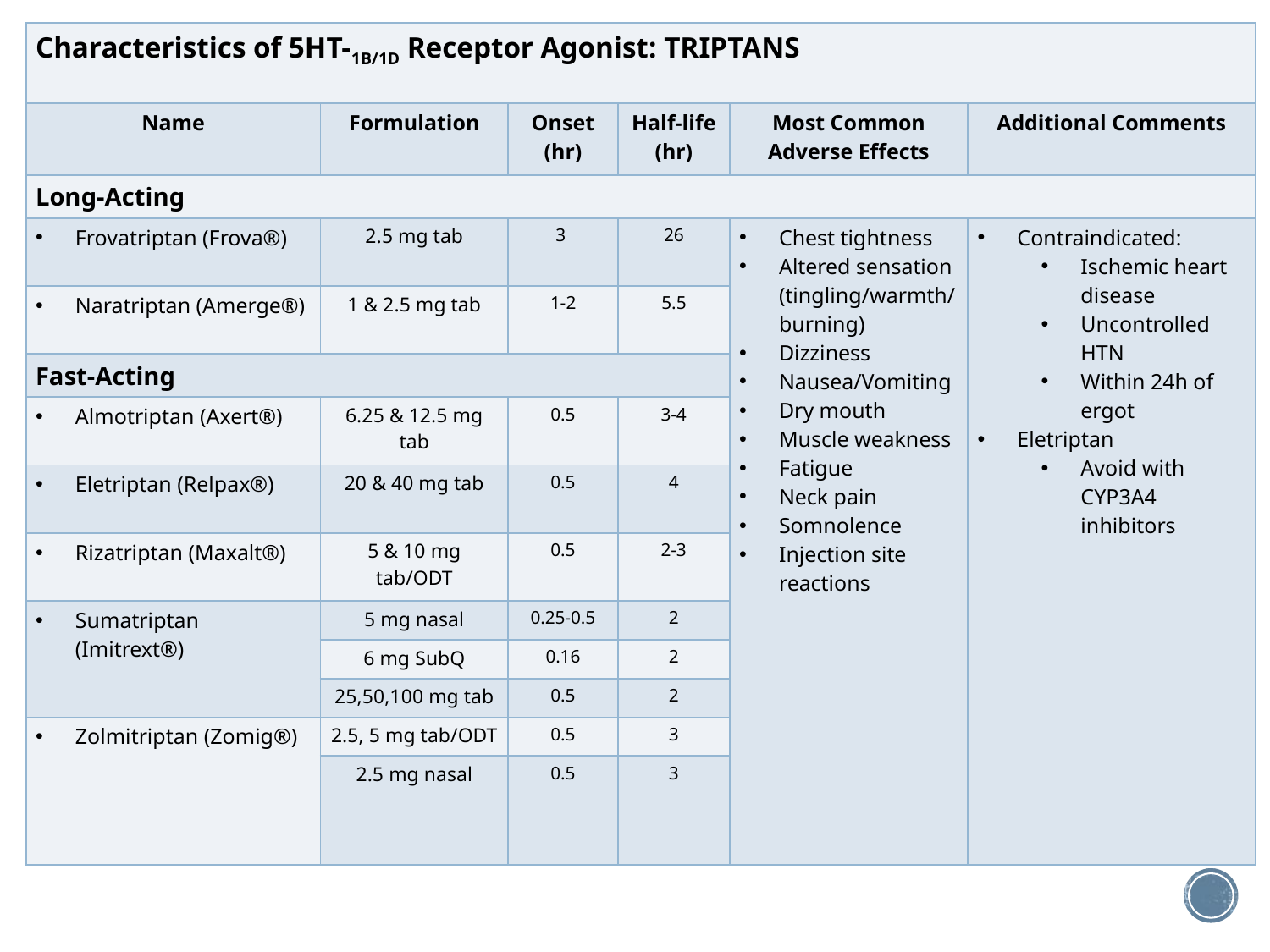

| Characteristics of 5HT-1B/1D Receptor Agonist: TRIPTANS | | | | | |
| --- | --- | --- | --- | --- | --- |
| Name | Formulation | Onset (hr) | Half-life (hr) | Most Common Adverse Effects | Additional Comments |
| Long-Acting | | | | | |
| Frovatriptan (Frova®) | 2.5 mg tab | 3 | 26 | Chest tightness Altered sensation (tingling/warmth/burning) Dizziness Nausea/Vomiting Dry mouth Muscle weakness Fatigue Neck pain Somnolence Injection site reactions | Contraindicated: Ischemic heart disease Uncontrolled HTN Within 24h of ergot Eletriptan Avoid with CYP3A4 inhibitors |
| Naratriptan (Amerge®) | 1 & 2.5 mg tab | 1-2 | 5.5 | | |
| Fast-Acting | | | | | |
| Almotriptan (Axert®) | 6.25 & 12.5 mg tab | 0.5 | 3-4 | | |
| Eletriptan (Relpax®) | 20 & 40 mg tab | 0.5 | 4 | | |
| Rizatriptan (Maxalt®) | 5 & 10 mg tab/ODT | 0.5 | 2-3 | | |
| Sumatriptan (Imitrext®) | 5 mg nasal | 0.25-0.5 | 2 | | |
| | 6 mg SubQ | 0.16 | 2 | | |
| | 25,50,100 mg tab | 0.5 | 2 | | |
| Zolmitriptan (Zomig®) | 2.5, 5 mg tab/ODT | 0.5 | 3 | | |
| | 2.5 mg nasal | 0.5 | 3 | | |

## Slide 22
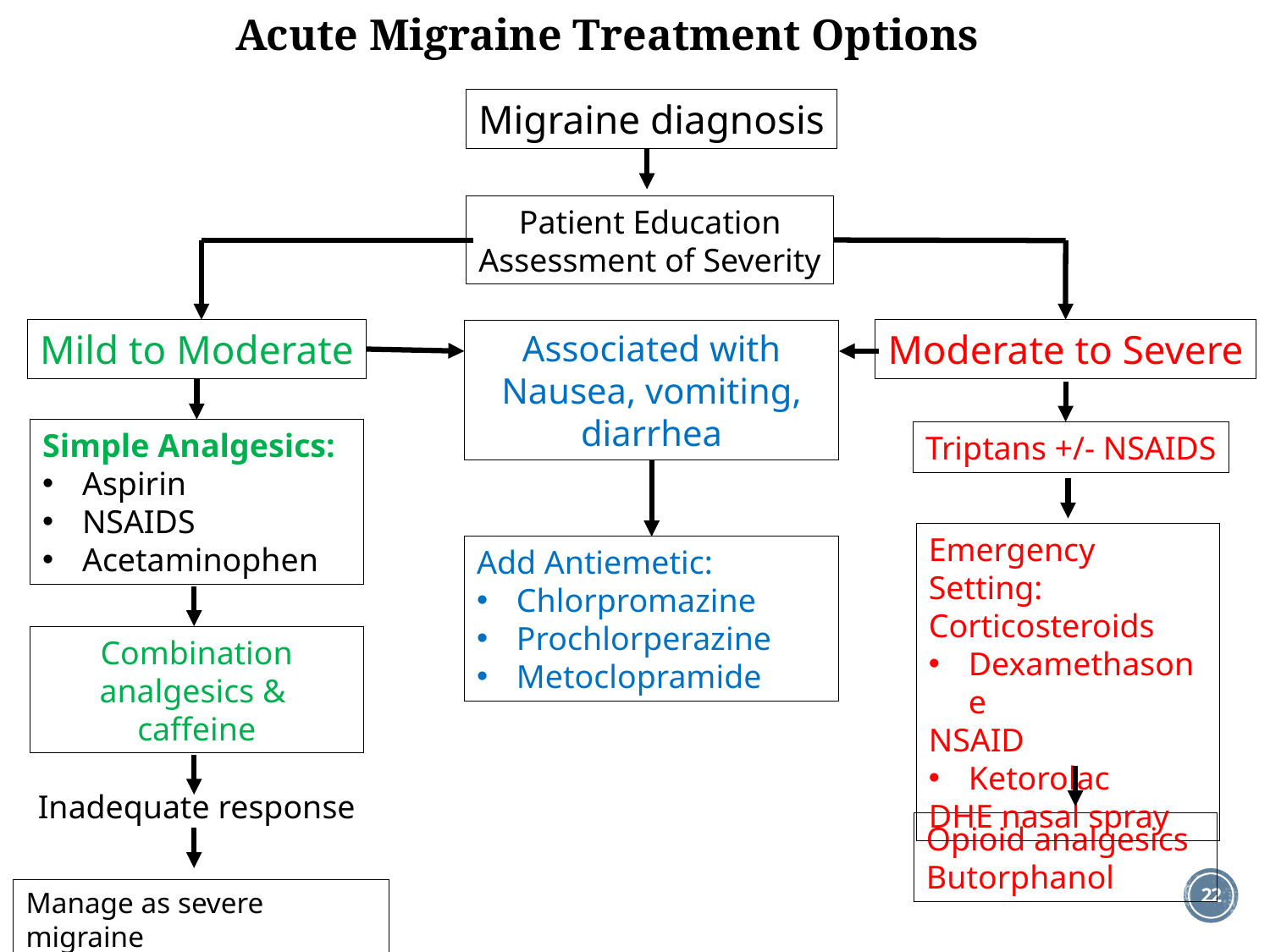

# Acute Migraine Treatment Options
Migraine diagnosis
Patient Education
Assessment of Severity
Mild to Moderate
Moderate to Severe
Associated with
Nausea, vomiting, diarrhea
Simple Analgesics:
Aspirin
NSAIDS
Acetaminophen
Triptans +/- NSAIDS
Emergency Setting:
Corticosteroids
Dexamethasone
NSAID
Ketorolac
DHE nasal spray
Add Antiemetic:
Chlorpromazine
Prochlorperazine
Metoclopramide
Combination analgesics &
caffeine
Inadequate response
Opioid analgesics
Butorphanol
22
Manage as severe migraine

## Slide 23
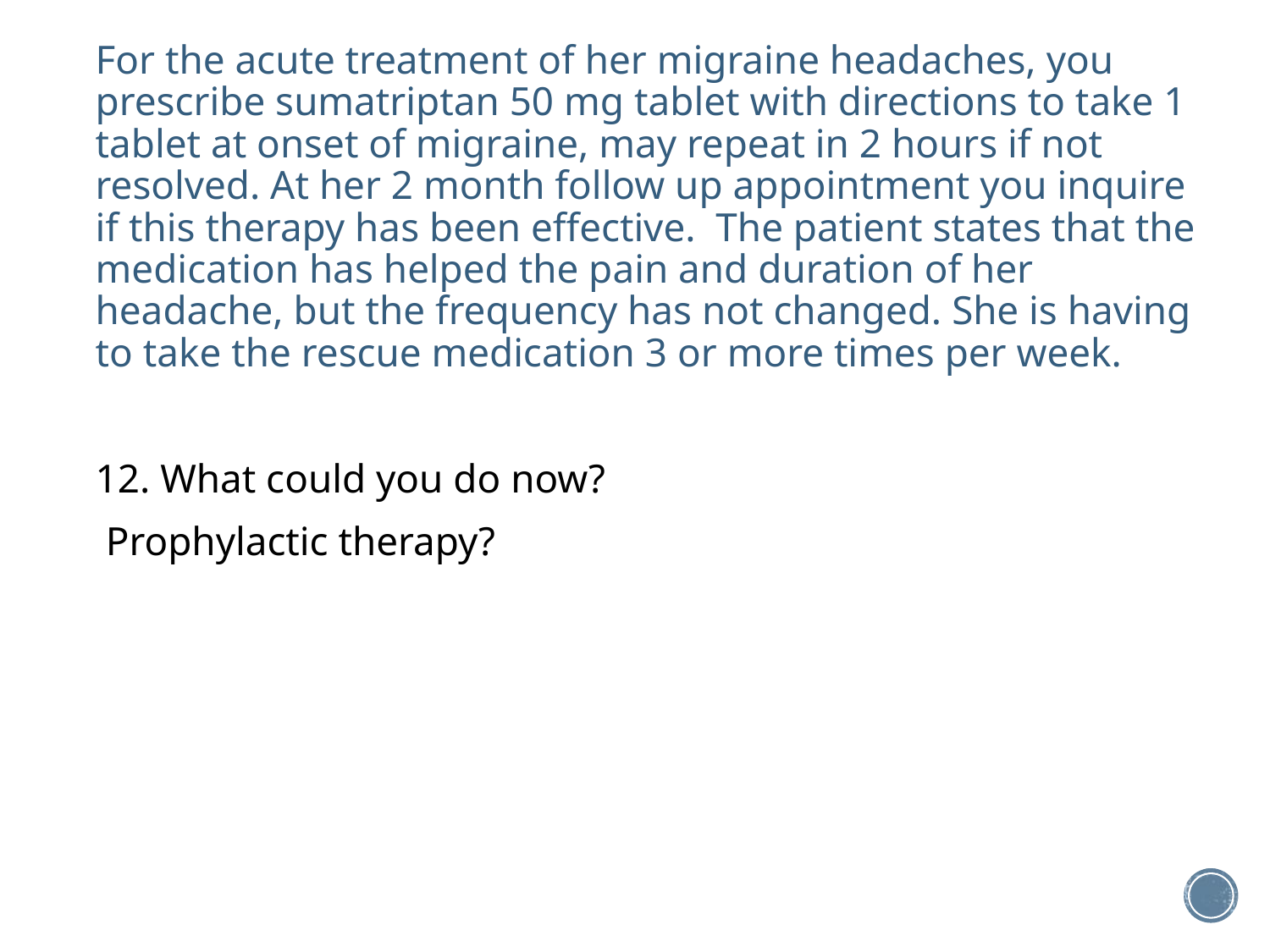

For the acute treatment of her migraine headaches, you prescribe sumatriptan 50 mg tablet with directions to take 1 tablet at onset of migraine, may repeat in 2 hours if not resolved. At her 2 month follow up appointment you inquire if this therapy has been effective. The patient states that the medication has helped the pain and duration of her headache, but the frequency has not changed. She is having to take the rescue medication 3 or more times per week.
12. What could you do now?
 Prophylactic therapy?

## Slide 24
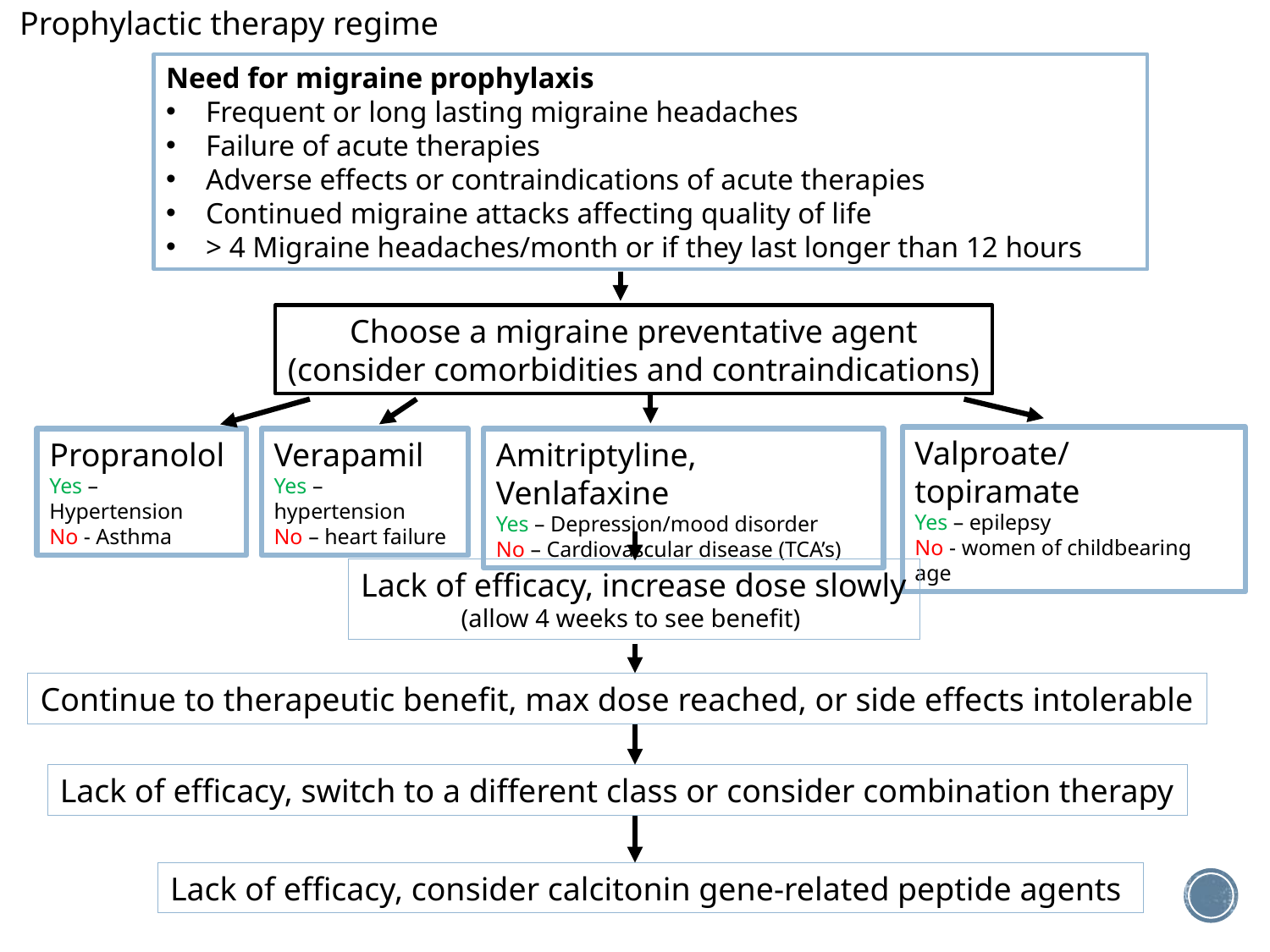

Prophylactic therapy regime
Need for migraine prophylaxis
Frequent or long lasting migraine headaches
Failure of acute therapies
Adverse effects or contraindications of acute therapies
Continued migraine attacks affecting quality of life
> 4 Migraine headaches/month or if they last longer than 12 hours
Choose a migraine preventative agent
(consider comorbidities and contraindications)
Valproate/topiramate
Yes – epilepsy
No - women of childbearing age
Propranolol
Yes – Hypertension
No - Asthma
Verapamil
Yes – hypertension
No – heart failure
Amitriptyline, Venlafaxine
Yes – Depression/mood disorder
No – Cardiovascular disease (TCA’s)
Lack of efficacy, increase dose slowly
(allow 4 weeks to see benefit)
Continue to therapeutic benefit, max dose reached, or side effects intolerable
Lack of efficacy, switch to a different class or consider combination therapy
Lack of efficacy, consider calcitonin gene-related peptide agents

## Slide 25
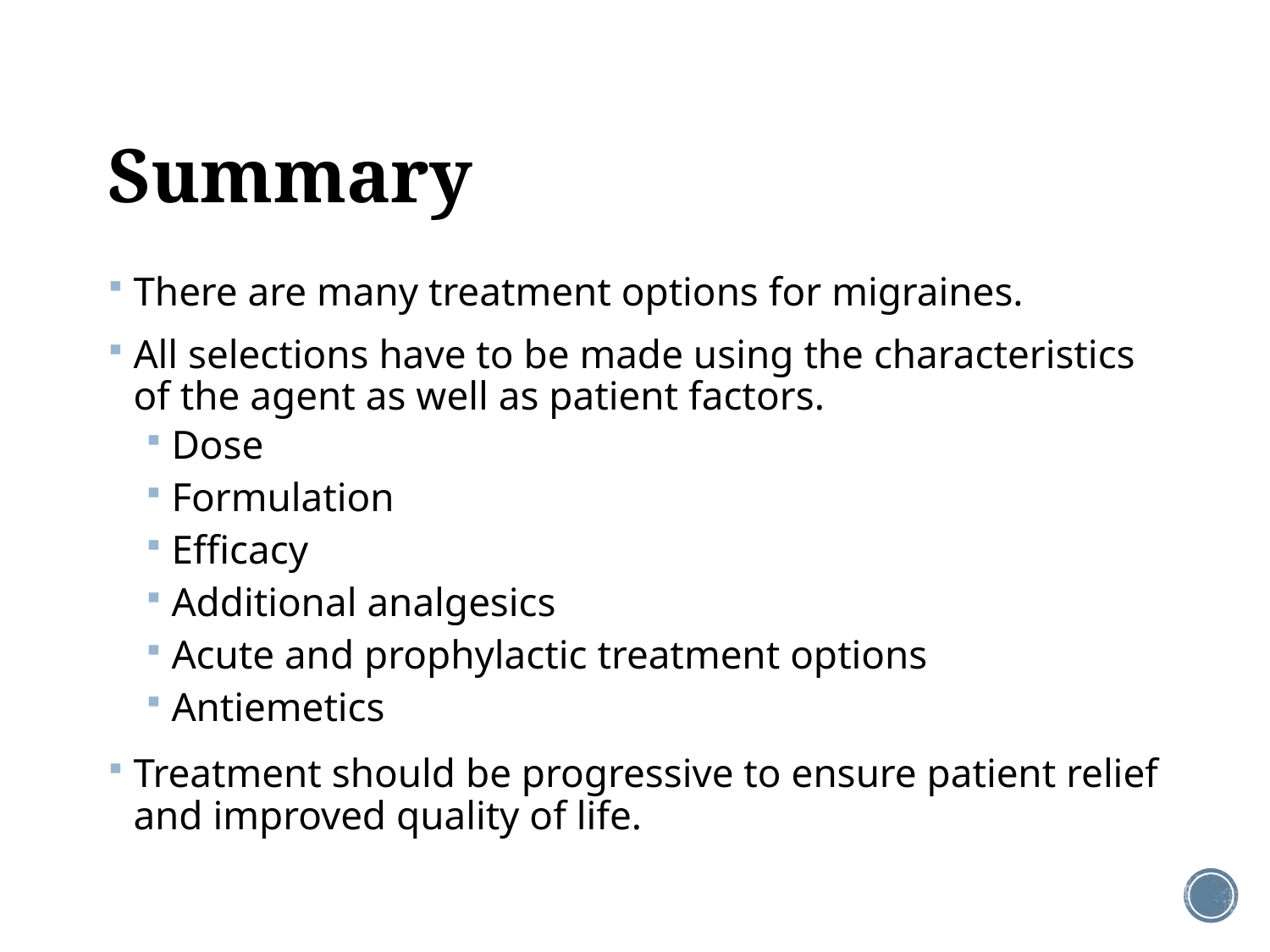

# Summary
There are many treatment options for migraines.
All selections have to be made using the characteristics of the agent as well as patient factors.
Dose
Formulation
Efficacy
Additional analgesics
Acute and prophylactic treatment options
Antiemetics
Treatment should be progressive to ensure patient relief and improved quality of life.

## Slide 26
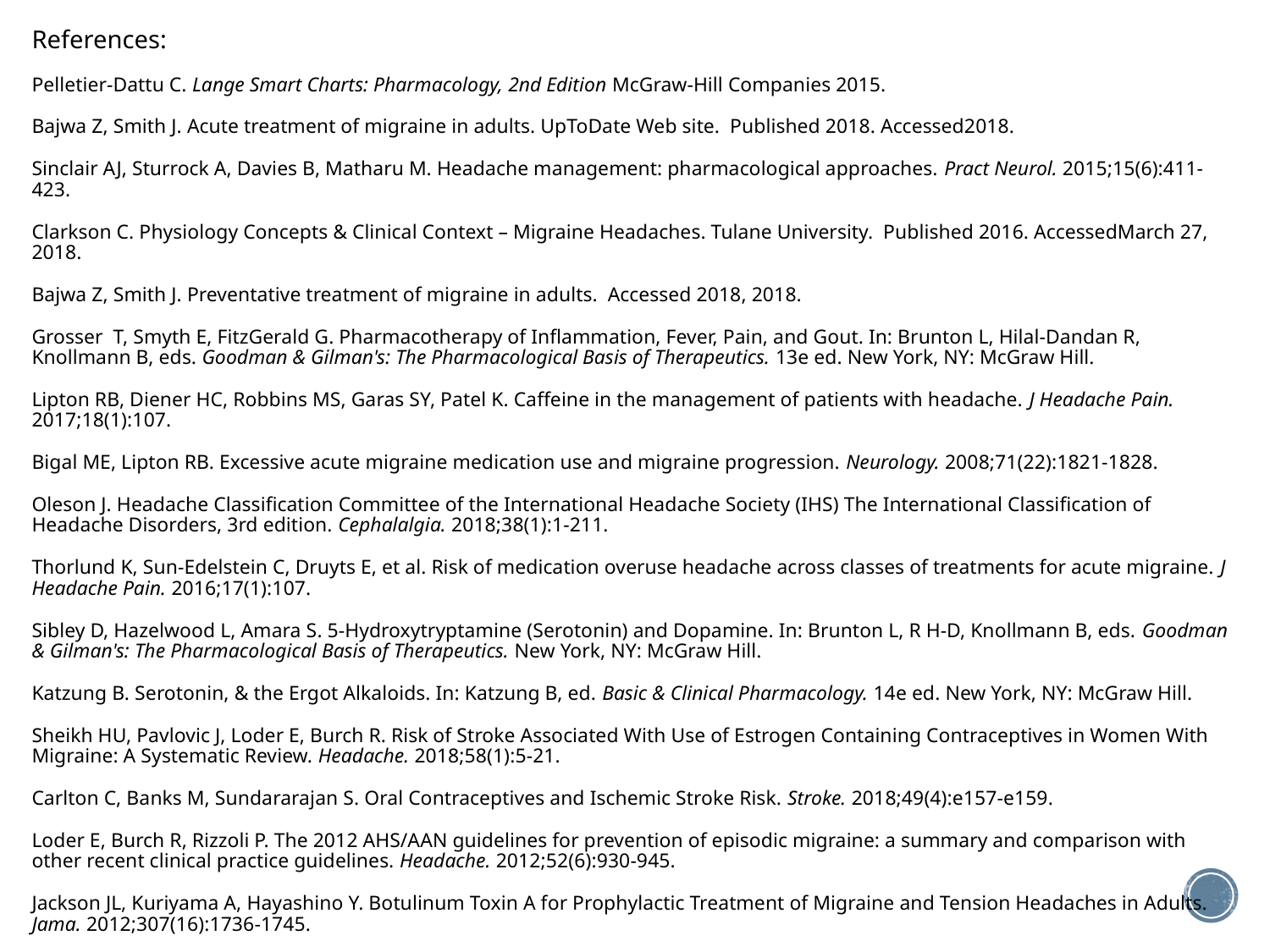

References:
Pelletier-Dattu C. Lange Smart Charts: Pharmacology, 2nd Edition McGraw-Hill Companies 2015.
Bajwa Z, Smith J. Acute treatment of migraine in adults. UpToDate Web site. Published 2018. Accessed2018.
Sinclair AJ, Sturrock A, Davies B, Matharu M. Headache management: pharmacological approaches. Pract Neurol. 2015;15(6):411-423.
Clarkson C. Physiology Concepts & Clinical Context – Migraine Headaches. Tulane University. Published 2016. AccessedMarch 27, 2018.
Bajwa Z, Smith J. Preventative treatment of migraine in adults. Accessed 2018, 2018.
Grosser T, Smyth E, FitzGerald G. Pharmacotherapy of Inflammation, Fever, Pain, and Gout. In: Brunton L, Hilal-Dandan R, Knollmann B, eds. Goodman & Gilman's: The Pharmacological Basis of Therapeutics. 13e ed. New York, NY: McGraw Hill.
Lipton RB, Diener HC, Robbins MS, Garas SY, Patel K. Caffeine in the management of patients with headache. J Headache Pain. 2017;18(1):107.
Bigal ME, Lipton RB. Excessive acute migraine medication use and migraine progression. Neurology. 2008;71(22):1821-1828.
Oleson J. Headache Classification Committee of the International Headache Society (IHS) The International Classification of Headache Disorders, 3rd edition. Cephalalgia. 2018;38(1):1-211.
Thorlund K, Sun-Edelstein C, Druyts E, et al. Risk of medication overuse headache across classes of treatments for acute migraine. J Headache Pain. 2016;17(1):107.
Sibley D, Hazelwood L, Amara S. 5-Hydroxytryptamine (Serotonin) and Dopamine. In: Brunton L, R H-D, Knollmann B, eds. Goodman & Gilman's: The Pharmacological Basis of Therapeutics. New York, NY: McGraw Hill.
Katzung B. Serotonin, & the Ergot Alkaloids. In: Katzung B, ed. Basic & Clinical Pharmacology. 14e ed. New York, NY: McGraw Hill.
Sheikh HU, Pavlovic J, Loder E, Burch R. Risk of Stroke Associated With Use of Estrogen Containing Contraceptives in Women With Migraine: A Systematic Review. Headache. 2018;58(1):5-21.
Carlton C, Banks M, Sundararajan S. Oral Contraceptives and Ischemic Stroke Risk. Stroke. 2018;49(4):e157-e159.
Loder E, Burch R, Rizzoli P. The 2012 AHS/AAN guidelines for prevention of episodic migraine: a summary and comparison with other recent clinical practice guidelines. Headache. 2012;52(6):930-945.
Jackson JL, Kuriyama A, Hayashino Y. Botulinum Toxin A for Prophylactic Treatment of Migraine and Tension Headaches in Adults. Jama. 2012;307(16):1736-1745.

## Slide 27
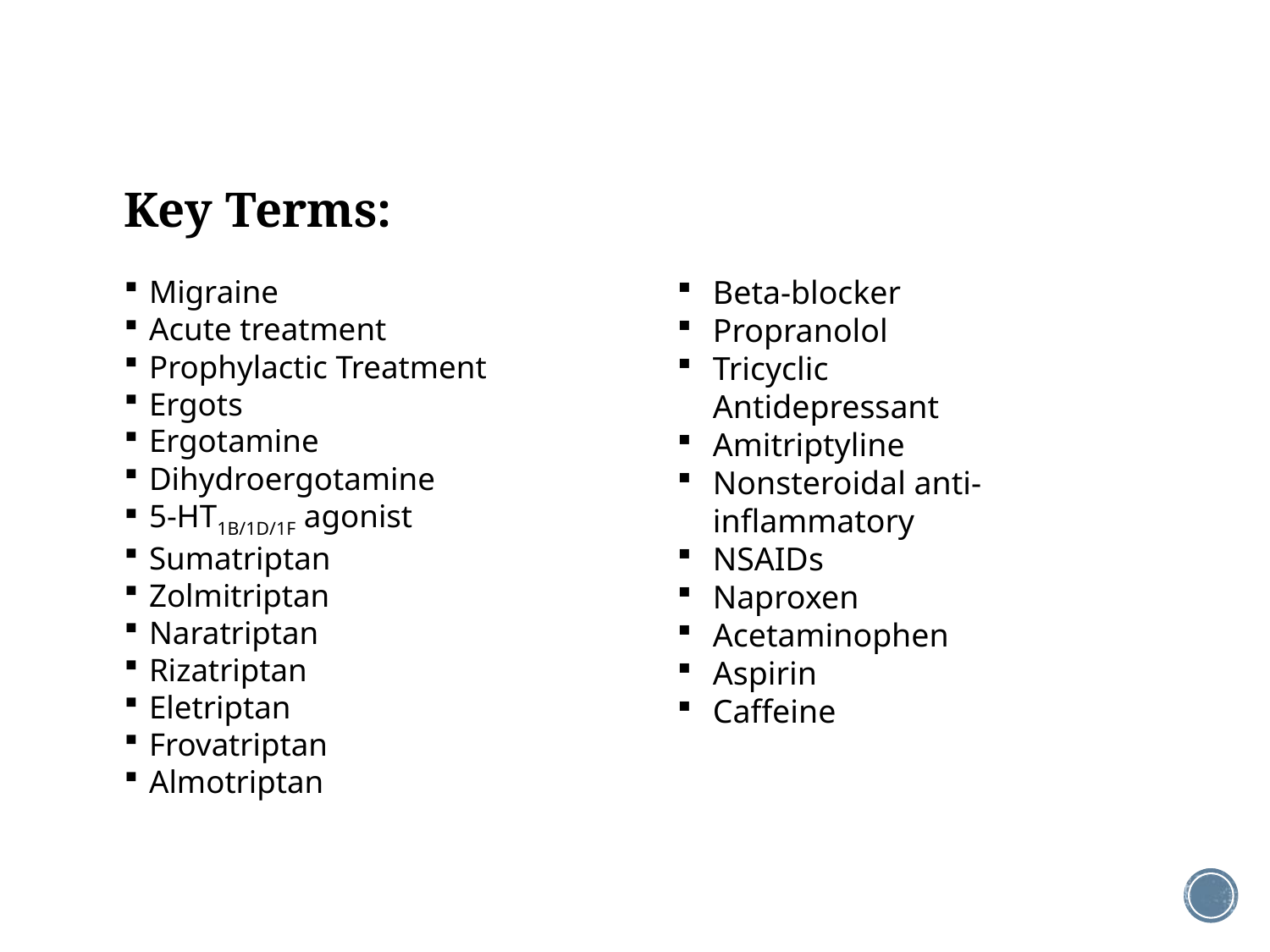

# Key Terms:
Beta-blocker
Propranolol
Tricyclic Antidepressant
Amitriptyline
Nonsteroidal anti-inflammatory
NSAIDs
Naproxen
Acetaminophen
Aspirin
Caffeine
Migraine
Acute treatment
Prophylactic Treatment
Ergots
Ergotamine
Dihydroergotamine
5-HT1B/1D/1F agonist
Sumatriptan
Zolmitriptan
Naratriptan
Rizatriptan
Eletriptan
Frovatriptan
Almotriptan
